# Physical exercise and its effects on people with Parkinson’s disease: Umbrella review

**DOI:** 10.1371/journal.pone.0293826

**Published:** 2023-11-02

**Authors:** Cristiano Padilha, Renan Souza, Fernando Schorr Grossl, Ana Paula Maihack Gauer, Clodoaldo Antônio de Sá, Sinval Adalberto Rodrigues-Junior

**Affiliations:** Health Sciences Post-Graduate Program, Universidade Comunitária da Região de Chapecó–Unochapecó, Chapecó, Santa Catarina, Brazil; University of Campania Luigi Vanvitelli: Universita degli Studi della Campania Luigi Vanvitelli, ITALY

## Abstract

**Introduction:**

Parkinson’s disease is neurodegenerative, complex and progressive, manifesting in a slow and irreversible way. Physical exercise has been proposed as therapeutic alternative to people with Parkinson´s disease.

**Objective:**

To synthesize knowledge about the effects of physical exercise on people with Parkinson´s Disease as presented by published systematic reviews.

**Methods:**

Nine electronic databases and two grey literature databases were searched for systematic reviews reporting the effects of physical exercises on people with Parkinson´s Disease. Searches involved a two-phase process, by, at least, two independent reviewers. Methodological quality of the included systematic reviews was assessed using AMSTAR-2.

**Results:**

From 2,122 systematic reviews, 139 were included. Motor outcomes were assessed in 91% of the studies, with balance being the most studied. Non-motor outcomes were assessed in 68% of the studies, with emphasis on quality of life. Physical exercises were classified into five categories: aerobic exercises, strength, combined, sensorimotor activities and other activity protocols. Findings of the systematic reviews suggest that all exercise categories can be prescribed to improve balance and mobility, while combined exercises, strength, and specific activities improve both motor and non-motor outcomes, and aerobic exercise and sensorimotor activities improve motor outcomes.

**Conclusion:**

Current evidence from systematic reviews suggests that physical exercises impacts both motor and non-motor outcomes in people with Parkinson´s Disease. Limits in evidence provided by the systematic reviews were related to methodological issues and to the description of the interventions and must be considered to improve decision-making and clinical application.

## Introduction

Neurological disorders are the main cause of morbidities and functional disability in the world [1]. Parkinson´s disease (PD) is one of the most common neurological disorders, along with Alzheimer’s disease (AD), Huntington’s disease, amyotrophic lateral sclerosis and frontotemporal dementia [2]. These diseases may vary in their pathophysiology, but they have in common their association with population aging, and the common presence of aggregated protein forms in the brain of affected individuals [3].

PD presents important motor symptoms, namely bradykinesia, muscle rigidity, bent posture, motor blocking, postural instability and tremor. The level of functional disability may be determined by the 5-point Hoehn and Yahr scale, which consider stages 1 to 3 as minimally disabled, meaning that they are still able to live independently. Stages 4 and 5, on the other hand, characterize severely disabled people and has been associated with highly compromised neurocognitive issues [4,5]. Physical impairment caused by PD may be potentiated by sarcopenia (loss of muscle mass) and osteoporosis (loss of bone mass), conditions common to old people that often coexist [6].

According to the Global Burden of Diseases [1], PD is the fastest growing, as the population ages and life expectancy increases. In 2016, PD caused more than 211 thousand deaths (93.5 thousand women and 117.5 thousand men), in addition to 3.2 million cases of functional disability (1.4 million women and 1 .8 million in men) [1]. Expectations are that the number of individuals with PD, as well as the duration of the disease will continue to increase, demanding effective prevention and treatment strategies [7].

In general, physical exercises have been proposed as an efficient intervention in the treatment of several chronic conditions, with positive responses on blood pressure [8], prevention and treatment of diabetes [9], improvement of lipoprotein profile, increase of insulin sensitivity, help on weight control [10], prevention and improvement of mild conditions of depressive disorders, anxiety, dyspnea, and quality of life [11], improvement of physical fitness, cognitive functioning, and mind-body connection [12] and production of neurogenesis and neuroprotection [13]. Although there is no cure for PD, exercise protocols involving gait training, balance and muscle strengthening have shown important effects on the physical capacity of patients [14], being considered a safe and effective approach [15].

Regular physical exercise, especially aerobic exercise is beneficial for patients with PD, as it reduces hypokinesia, bradykinesia, gait disturbances, neuronal degeneration, loss of independence to perform activities of daily living (ADLs) and maintains the cardiovascular capacity in individuals classified with mild and moderate PD [16]. The literature presents dozens of systematic reviews (SR) that analyze the effect of physical exercise in people with PD. Several types of exercises are found along with a diversity of parameters for the application of these exercises, which, in turn, can make their prescription difficult. There are several exercise protocols and an enormous variability of elements that make up the exercise dose, such as type, weekly frequency, volume, intensity, duration of intervention, among others, making it difficult to establish a more adequate protocol. Considering the complexity of this scenario, this umbrella review aimed to collect the evidence of exercise in PD.

## Methodology

### Study design

This study was first conceived as a scoping review of SR, therefore, an umbrella review [17], and had its protocol registered in Open Science Framework (https://osf.io/knjuq/). The research question was structured based on PCC acronym: *P (population)* = people with Parkinson´s disease; *C (concept)* = physical exercise; *C (context)* = global context/impact on motor and non-motor symptoms.

### Eligibility criteria and search

Systematic reviews of physical exercises as interventions to people with PD, regardless of the level of disability were considered eligible and were searched in the following electronic databases: Cochrane Library, CINAHL, EMBASE, PEDro, Medline via PubMed, LILACS via BVS, DARE, Scopus, SPORTDiscus and Web of Science. Grey literature was also searched in Grey Matters and national and international universities and research centers catalogs containing theses and dissertations. There was no restriction based on place (origin), language, date, and age/sex of participants. The references of included studies were also searched. Studies that did not fulfill the criteria for population, intervention and type of study were excluded.

The search strategy was built with the aid of an experienced librarian (CSS) using the MeSH terms (Medical Subject Headings) (https://www.ncbi.nlm.nih.gov/mesh/), DeCS (https://decs.bvsalud.org/) and EMTREE (https://www-embase.ez224.periodicos.capes.gov.br/emtree) as indexed terms, along with terms from natural language and Boolean operators to combine them. The search strategy was first built in PubMed and was repeated in the other databases respecting their own syntax rules, to ensure that no relevant study was lost. The search strategy was built from inception and the later search was performed on September 9^th^, 2022. Table 1 presents the search strategy developed for PubMed. The search strategies applied to the other databases may be found in S1 Table.

**Table 1:** Search strategy applied to PubMed database.

("Parkinson’s Disease" OR "Idiopathic Parkinson’s Disease" OR "Lewy Body Parkinson Disease" OR "Lewy Body Parkinson’s Disease" OR "Primary Parkinsonism" OR "Parkinsonism, Primary" OR "Parkinson Disease, Idiopathic" OR "Parkinson’s Disease" OR "Parkinson’s Disease, Idiopathic" OR "Parkinson’s Disease, Lewy Body" OR "Idiopathic Parkinson Disease" OR "Paralysis Agitans") AND (Exercise* OR "Physical Activity" OR "Activities, Physical" OR "Activity, Physical" OR "Physical Activities" OR "Exercise, Physical" OR "Exercises, Physical" OR "Physical Exercise" OR "Physical Exercises" OR "Acute Exercise" OR "Acute Exercises" OR "Exercise, Acute" OR "Exercises, Acute" OR "Exercise, Isometric" OR "Exercises, Isometric" OR "Isometric Exercises" OR "Isometric Exercise" OR "Exercise, Aerobic" OR "Aerobic Exercise" OR "Aerobic Exercises" OR "Exercises, Aerobic" OR "Exercise Training" OR "Exercise Trainings" OR "Training, Exercise" OR "Trainings, Exercise") AND ("systematic review" OR "systematic literature review" OR "systematic scoping review" OR "systematic narrative review" OR "systematic qualitative review" OR "systematic evidence review" OR "systematic meta-review" OR "systematic critical review" OR "systematic mixed studies review" OR "systematic mapping review" OR "systematic cochrane review" OR "systematic search and review" OR "systematic integrative review" OR "scoping review")

### Study selection

The studies were selected in a two-phase process. First, they were selected by reading of title and abstract. For that, the references databanks were uploaded to MyEndNote Desktop where duplicates were removed. Following, the resulting dataset was uploaded to Rayyan, where the first phase took place. The second phase involved full-text reading for selection. Both were conducted by two previously trained reviewers (CP and RS) and when there were divergencies, they were solved by consensus involving two other reviewers (CAS and SARJ).

### Data extraction

Data were extracted from the studies independently by four reviewers (CP, RS, FGS and APG) and filled in a previously prepared Excel worksheet (Microsoft Excel 2013, Microsoft Corporation), which was tested and revised by other researchers (CAS and SARJ). The following information was extracted: regarding the studies (authors, year of publication, country of origin, title, purpose, study design, conclusions), the population (sample size, age, sex, diagnostic classification), related to the interventions (type of training, session duration, training time, intensity, frequency and control groups), outcomes (motor and non-motor outcomes, outcome measures and main results). The methodological quality of the study was assessed by four reviewers (CP, RS, FGS and APG) using the A MeaSurement Tool to Assess systematic Reviews (AMSTAR-2). AMSTAR 2 is a 16-item, domain-based instrument meant to critically appraise systematic reviews of randomized controlled trials [18]. Seven domains of the instrument are considered critical to rate the methodological quality of the study, namely protocol registration before commencement of the review, adequacy of the literature search, justification for excluding individual studies, risk of bias from individual studies included in the review, appropriateness of meta-analytical methods, consideration of risk of bias when interpreting the results of the review and assessment of the likely impact of publication bias [18,19]. The identification of weaknesses in these critical domains allows one to correctly interpret the information provided by the systematic reviews, and provides four classification levels: ‘high quality’, ‘moderate quality’, ‘low quality’ and ‘critically low quality’ evidence [18].

### Synthesis

Data were synthetized based on descriptive mapping of the breadth of research on the issue and a narrative summary.

## Results

**Fig 1:**
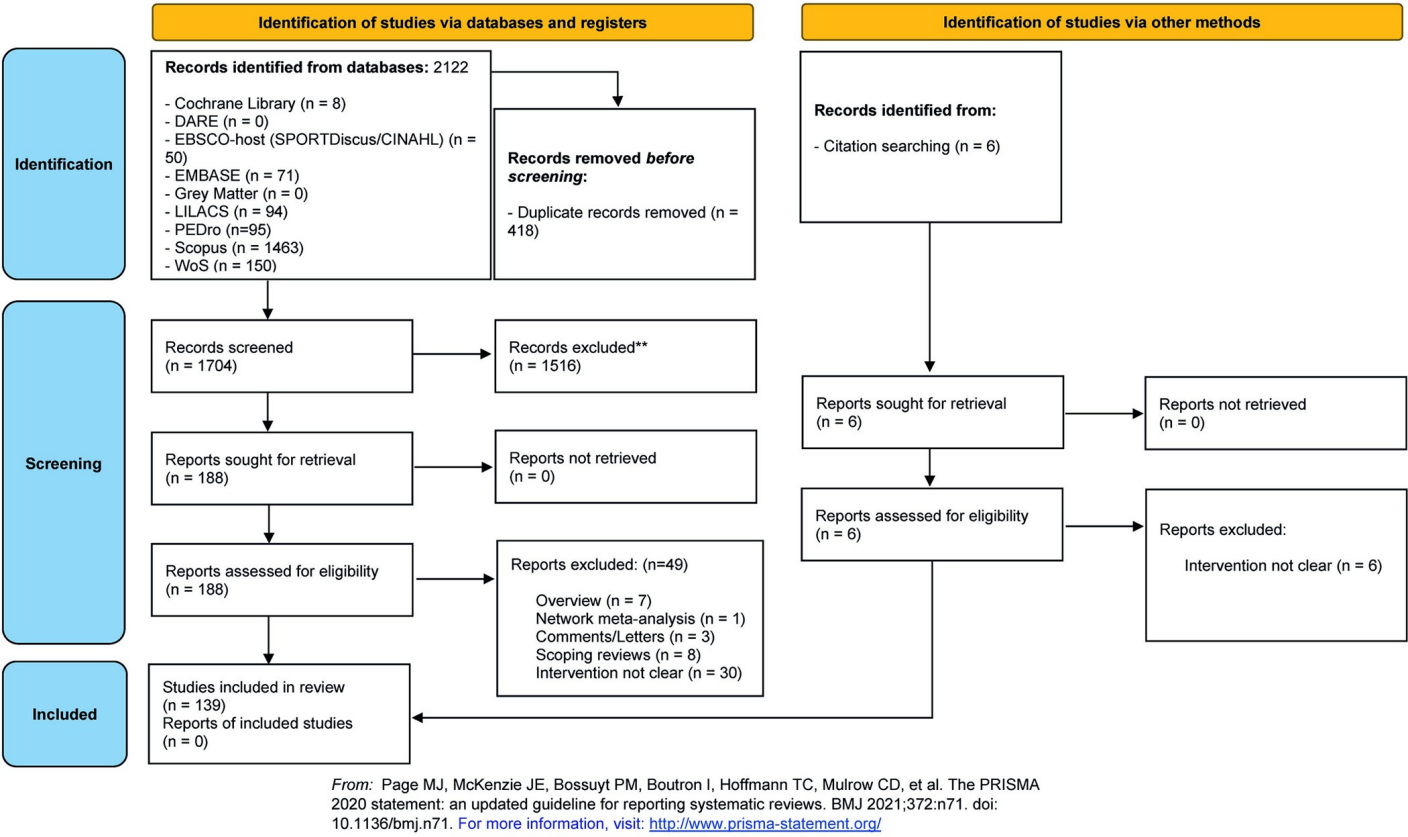
Study flow diagram.

The study flow diagram is presented in Fig 1. Database records totalized 2,122 references, which were reduced to 1704 studies after duplicate removal. A total of 188 studies were analyzed by full-text reading from which 49 studies were excluded. The reasons for exclusion are depicted in the study flow diagram. Finally, 139 studies were included and had data extracted for the review.

A significant number of physical exercise interventions was identified in the included systematic reviews and was categorized into five distinct categories: aerobic exercise, strength exercise, combined exercise, sensory-motor activities and other protocols. These categories are described in Table 2, along with the interventions classified in each category.

**Table 2:** Categorization of the physical exercises identified.

Category	Physical exercises
AEROBIC EXERCISE: Involves movements of the large muscles of the body in a rhythmic manner for prolonged periods [20]	Cycling, treadmill, walking, Qigong, cross training, rowing ergometer, robot-assisted gait training, Nordic walking, endurance, rhythm retraining, treadmill walking, HIIT-LOW on treadmill, resistance training, elliptic, bike exercise, jogging, gait training with Body Weight Support (BWS), interval training, forced cycling exercise, Sitting and Standing; functional exercise; circuit training, vigorous intensity exercise, split belt treadmill (SBT).
STRENGTH EXERCISE: Activities in which muscles work or hold against an applied force or weight to improve muscle fitness (i.e., functional parameters of strength, endurance, and power) [20]	Whole body resistance training, strengthening exercises, functional training, Power Yoga, resistance training intensity, machine, weight training, strength training, lower or whole-body strength, resistance training program, Progressive Resistance Training (PRT), Inspiratory Muscle Strength Training (IMST), Expiratory Muscle Strength Training (EMST).
COMBINED EXERCISE: Consists of planned activities, structured and repetitive body movements that are performed to improve or maintain one or more components of physical fitness [20]	Walking training, strength, flexibility, balance, aerobic, agility, virtual reality, Tango, breathing, bodyweight supported, Tai Chi, Qigong, Brazilian samba, resistance, stretching, physiotherapy, Hatha Yoga, functional bodyweight, power, Pilates, Movement Strategy Group, dance, high-intensity physical training, ParkFit, treadmill training, stationary bike, cycling, rowing, multimodal exercises, coordination, motor therapy, respiratory muscle strength training, exergaming, LSVT BIG, boxing, task-specific training.
SENSORY-MOTOR ACTIVITIES: Combine exercises with interactions of emotional, mental, social, spiritual and behavioral factors directly affect our health. Mind-body interventions increase the body’s self-awareness, thereby increasing energy, mental clarity, concentration, and an individual’s ability to tolerate physical discomfort [21]	Neuro Proprioceptive Function, Qigong, Tai Chi, virtual reality, Exergame (WII, KINETIC, WIImove), mind-body, Yoga, LSVT-BIG, Pilates, Traditional Chinese Medical Exercise.
OTHER PROTOCOLS: Exercises that do not have volume and intensity control, or which are not classified as previous exercises (i.e. aquatic exercises, dance or any other physical activities)	Aquatic exercise, aerobic capacity, balance and postural control, mobility and strength, whole body stretching, cardiovascular activities and relaxation, resistance exercise, water walking, aquatic therapeutic exercise, hydrotherapy. Tango, Waltz, Foxtrot, Contemporary dance, Irish dance, Modern dance, PD-specific dance, Tango-based dance, Ballet, Music-based Movement therapy (MbM), improvisation, American Ballroom, RGRhythm, Jazz, Turo Dance. Exercise and motor training, highly challenging balance training, cueing, exercise training, task training (motion strategy), strength training, stretching exercise/ROM, ground walking/exercise, treadmill walking, dance, hydrotherapy, Tai Chi, virtual reality, mental practice, aquatic exercise, aerobic exercise, robotic gait training, boxing, whole body vibration, Nordic walking training, cardiovascular warm-up, functional exercises, treadmill using external auditory cues, Occupational Therapy (TO), walking, aerobic training, cycling, LSVT, Qigong; progressive resistance exercise training, Tango, home-exercise program; physiotherapy, Ronnie Gardiner Rhythm, Tai Chi; Nordic walking, body weight support, dual task training, whole body, vibration, Qigong, home-based, mobility, multi-component exercise program (home), cycling, training program involving daily activities, sensory therapy with external cues, therapy physical, boxing, multimodal, WII, goal-based exercise, proprioceptive, dual-task, virtual reality balance training, LSVT-BIG, breathing exercises, enhanced exercise therapy for PD (EXCEED).

Table 3 presents the characteristics of participants, interventions and outcomes involved in the systematic reviews. Complementary characterization of these elements is found in S2 Table. Twenty-two studies published from 2009 to 2022 addressed the effect of aerobic exercises on seventeen different outcomes. Thirteen studies (2013:2021) accounted for the effect of strength exercises on fourteen outcomes. Nineteen studies (2014:2021) were dedicated to the effect of combined exercises on seventeen outcomes. Thirty studies published from 2008 to 2022 synthesized the effect of sensory-motor activities on seventeen outcomes. Fifty-four studies, from 2005 to 2022, addressed the effect of other exercise protocols on seventeen outcomes.

**Table 3:** Summary of participants characteristics, interventions and outcomes.

Type of exercise intervention	Number of reporting SR (%)	Lower count	Higher count
***Aerobic***			
Time range (year:year)	22 (100%)	2009	2022
Number of RCT	22 (100%)	3	27
Number of participants	22 (100%)	44	1,210
Age range (years)	18 (81.8%)	30	80
PD level (H & Y– 1–4 scale)	19 (86.4%)	1	4
Duration range (years)	14 (63.6%)	0.3	18.1
Session time (min)	22 (100%)	2	90
Duration (weeks)	21 (95.5%)	1	64
Frequency (days)	19 (86.4%)	1	7
Intensity	21 (95.5%)	SF*	SF
Control group	19 (86.4%)	SF	SF
Outcomes assessed	22 (100%)	QOL; FAT; BAL; MOV; MOT; MOB; FALL; CARD; WLK; COG; ANX/DEP; BDNF; ADL; DIS; APA; STR; HUM	
***Strength***			
Time range (year:year)	13 (100%)	2013	2021
Number of RCT	13 (100%)	4	31
Number of participants	13 (100%)	92	1,239
Age range (years)	12 (92.3%)	45	90
PD level (H & Y– 1–4 scale)	10 (76.9%)	1	4
Duration range (years)	6 (46.2%)	1.1	12.3
Session time (min)	11 (84.6%)	15	90
Duration (weeks)	13 (100%)	1	104
Frequency (days)	12 (92.3%)	1	6
Intensity	11 (84.6%)	SF	SF
Control group	12 (92.3%)	SF	SF
Outcomes assessed	22 (100%)	MOT; WLK; STR; MOB; CARD; QOL; BAL; HUM; MOV; FLX; COG; FAT; FALL; BRE	
***Combined***			
Time range (year:year)	19 (100%)	2014	2021
Number of RCT	19 (100%)	9	50
Number of participants	19 (100%)	79	2,972
Age range (years)	17 (89.5%)	29	89
PD level (H & Y– 1–4 scale)	17 (89.5%)	1	4
Duration range (years)	11 (57.9%)	0.1	13.3
Session time (min)	19 (100%)	15	120
Duration (weeks)	19 (100%)	2	104
Frequency (days)	19 (100%)	1	7
Intensity	17 (89.5%)	SF	SF
Control group	19 (100%)	SF	SF
Outcomes assessed	19 (100%)	QOL; COG; ANX/DEP; BAL; MOV; MOT; FALL; STR; CARD; ADL; WLK; HUM; SLP; BDNF; DIS; BRE; MOB	
***Sensory-motor activities***			
Time range (year:year)	31 (100%)	2008	2022
Number of RCT	31 (100%)	4	35
Number of participants	27 (87.1%)	84	1,210
Age range (years)	27 (87.1%)	40	86
PD level (H & Y– 1–4 scale)	24 (77.4%)	1	4
Duration range (years)	9 (29.0%)	2	10
Session time (min)	26 (83.9%)	5	120
Duration (weeks)	29 (93.6%)	1	52
Frequency (days)	29 (93.6%)	1	7
Intensity	4 (12.9%)	SF	SF
Control group	29 (93.6%)	SF	SF
Outcomes assessed	31 (100%)	QOL; ANX/DEP; BAL; MOV; MOT; FALL; CARD; ADL; MOB; WLK; STR; COG; BRE; FLX; HUM; SLP; WLB	
***Other protocols***			
Time range (year:year)	54 (100%)	2005	2022
Number of RCT	54 (100%)	2	191
Number of participants	54 (100%)	37	7,998
Age range (years)	48 (88.9%)	18	90
PD level (H & Y– 1–4 scale)	43 (79.6%)	1	4
Duration range (years)	16 (29.6%)	0.3	18.7
Session time (min)	49 (90.7%)	15	135
Duration (weeks)	53 (98.2%)	2	104
Frequency (days)	53 (98.2%)	1	7
Intensity	3 (5.6%)	SF	SF
Control group	48 (88.9%)	SF	SF
Outcomes assessed	54 (100%)	BAL; MOV; WLK; MOB; STR; ADL; FLX; ANX/DEP; MOT; CARD; FALL; COG; DIS; HUM; FAT; SLP; APA	

NR–not reported.

QOF–Quality of life; FAT–Fatigue; BAL–Balance; MOV–Movement/walking; MOT–Motor function; FALL–Falls; CARD–cardiorespiratory; MOB–mobility; WLK–walking; COG–cognitive function; ANX/DEP–anxiety/depression; BNDF–brain-derived neurotrophic factor; ADL–activities of daily living; DIS–disease severity; APA–apathy; STR–strength; HUM–humor; SLP–sleep; WLB–well-being; FLX–flexibility.

*SF–Supplementary file.

Information of participants such as age range, PD level and duration were available in most, but not all studies. The duration, frequency, intensity and the presence of a control group were also missing in some studies, regardless of the exercise intervention being assessed.

Table 4 indicates the non-motor and motor outcomes improved by the physical exercises and the AMSTAR classification of the evidence provided by the systematic reviews to recommend them. S3 Table reveals the classification of each study considering the AMSTAR questions and domains.

**Table 4:** Non-motor and motor outcomes improved by the physical exercises and methodological quality of the systematic reviews.

Author, year	Non-motor outcomes	Motor outcomes	AMSTAR
AEROBIC EXERCISES			
Mehrholz, 2015 [15]	-	MOV; WLK	High
Robinson, 2019 [22]	-	MOB; WLK	High
Lorenzo Garcia, 2021 [23]	-	BAL; MOT; WLK	High
Mackay, 2017 [24]	BNDF	-	Moderate
Li, 2020 [25]	QOL	BAL; MOV	Moderate
Tiihonen, 2021 [26]	QOL	MOV; MOT	Moderate
De Almeida, 2022 [27]	-	MOT	Moderate
Shu, 2014 [28]	-	BAL; MOV; MOT	Low
Cascaes da Silva, 2016 [29]	QOL	BAL; MOV; MOT; MOB	Low
Alwadat, 2018 [30]	-	BAL; MOV; MOB	Low
De Santis, 2020 [31]	QOL; COG; HUM; FAT; APA	BAL; MOB; WLK	Low
Rodríguez, 2020 [32]	-	CARD	Low
Salse-Batán, 2022 [33]	QOL	MOV	Low
Herman, 2009 [34]	QOL	MOV; MOT	Critically low
Jambeau, 2011 [35]	-	MOT; MOB; WLK	Critically low
Lamotte, 2015 [36]	-	MOV; CARD	Critically low
Bombieri, 2017 [37]	QOL	MOV; MOB; WLK	Critically low
Flach, 2017 [38]	-	MOT	Critically low
Seuthe, 2019 [39]	-	MOV; WLK	Critically low
Aburub, 2020 [40]	-	CARD	Critically low
Miner, 2020 [41]	-	MOT; MOB	Critically low
Braz, 2021 [42]	-	MOV; MOB; STR	Critically low
STRENGTH EXERCISES			
Saltychev, 2016 [43]	-	WLK; STR; CARD	High
Lima, 2013 [44]	-	WLK; STR	Moderate
Cruickshank, 2015 [45]	-	MOT; MOB; STR	Moderate
Li, 2020 [46]	QOL	BAL; STR	Moderate
Brienesse, 2014 [47]	-	MOT; MOB; STR; CARD	Low
Chung, 2016 [48]	-	MOT; STR	Low
Roeder, 2015 [49]	-	STR	Low
Tillman, 2015 [50]	-	STR	Low
Ramazzina, 2017 [51]	QOL	STR	Low
Van de Wetering-van Dongen, 2020 [52]	QOL; BRE	STR	Low
Rodríguez, 2020 [53]	QOL; BRE	STR	Critically low
Braz, 2021 [54]	-	BAL; MOV; STR; CARD	Critically low
De Lima, 2022 [55]	QOL	BAL; STR	Critically low
COMBINED EXERCISES			
Choi, 2020 [56]	QOL	-	High
Cristini, 2021 [57]	SLP	-	High
Uhrbrand, 2015 [58]	-	STR; CARD	Moderate
Ni, 2018 [59]	-	BAL; MOV; WLK	Moderate
Stuckenschneider, 2019 [60]	COG	-	Moderate
Choi, 2020 [61]	-	BAL; MOV; MOT; WLK	Moderate
Johansson, 2020 [62]	BDNF	-	Moderate
McMahon, 2020 [63]	BRE	WLK	Moderate
Gilat, 2021 [64]	-	MOV	Moderate
Ruiz-Gonzalez, 2021 [65]	BDNF	-	Moderate
Da Silva, 2016 [28]	QOL	-	Low
Da Silva, 2018 [66]	COG	-	Low
Hirsch, 2018 [67]	BDNF	MOT	Low
Da Costa, 2020 [68]	QOL	BAL; MOT	Low
Gamborg, 2022 [69]	QOL; ANX/DEP	BAL; MOV; MOT; WLK; STR; CARD	Low
Tambosco, 2014 [70]	QOL	BAL; MOV; MOT; STR; CARD	Critically low
Reynolds, 2016 [71]	COG; HUM; SLP	-	Critically low
Smith, 2020 [72]	-	MOB	Critically low
Molina, 2021 [73]	-	MOT	Critically low
SENSORY-MOTOR ACTIVITIES			
Ni, 2014 [74]	-	BAL; MOB	High
Dockx, 2016 [75]	-	WLK	Moderate
Song, 2017 [76]	QOL; ANX/DEP	BAL; MOT; FALL; STR	Moderate
McDonell, 2018 [77]	-	MOV; MOT; WLK	Moderate
Liu, 2019 [78]	-	BAL; FALL; MOB	Moderate
Alexandre, 2020 [79]	-	MOV; WLK	Moderate
Chen, 2020 [80]	-	BAL; MOT; WLK	Moderate
Jin, 2019 [81]	QOL; ANX/DEP	BAL; MOT	Moderate
Yu, 2020 [82]	-	BAL; MOV; MOT	Moderate
Cugusi, 2021 [83]	QOL	-	Moderate
Elena, 2021 [84]	QOL	BAL; MOV	Moderate
Wang, 2021 [85]	QOL	BAL; MOV	Moderate
Yang, 2014 [86]	-	BAL; MOT; MOB	Low
Harris, 2015 [87]	-	BAL; MOT	Low
Yang, 2015 [88]	-	BAL; MOT	Low
Kwok, 2016 [20]	ANX/DEP	BAL; MOT; MOB; WLK; STR; CARD; ADL; FLX	Low
Winser, 2018 [89]	-	BAL; MOT; FALL	Low
Santos, 2019 [90]	QOL	BAL	Low
Suárez-Iglesias, 2019 [91]	-	BAL; MOB; STR; CARD	Low
Suárez-Iglesias, 2022 [92]	-	MOT	Low
Garcia-Lopez, 2021 [93]	-	BAL; FALL	Low
Lee, 2008 [94]	-	MOT; FALL	Critically low
Toh, 2013 [95]	-	-	Critically low
Zhou, 2015 [96]	-	BAL; MOT	Critically low
Cwiekala-Lewis, 2016 [97]	-	BAL; MOV; MOT; FALL; ADL; FLX	Critically low
Stickdorn, 2018 [98]	-	-	Critically low
Garcia-Agunde, 2019 [99]	COG	BAL; MOT	Critically low
Kamieniarz, 2020 [100]	QOL	BAL; MOV; FALL; WLK	Critically low
Mailankody, 2021 [101]	ANX/DEP	BAL	Critically low
Campo-Prieto, 2021 [102]	-	MOT	Critically low
Sevcenko, 2022 [103]	QOL; COG	BAL; MOV; MOT; MOB; ADL	Critically low
OTHER PROTOCOLS			
Perry, 2019 [104]	-	MOT	High
Cugusi, 2019 [105]	QOL	BAL; FALL	High
Pinto, 2019 [106]	-	BAL; MOB	High
Abou, 2021 [107]	-	FALL	High
Goodwin, 2008 [108]	QOL	BAL; MOV; MOB	Moderate
Allen, 2011 [109]	-	BAL	Moderate
Shen, 2015 [110]	-	BAL; MOV; FALL	Moderate
Wang, 2016 [111]	-	BAL; MOV	Moderate
Klamroth, 2016 [112]	-	BAL	Moderate
Delabary, 2017 [113]	-	MOT; MOB	Moderate
Flynn, 2019 [114]	-	BAL; MOV	Moderate
Kalyani, 2019 [115]	COG	MOV; WLK	Moderate
Zhang, 2019 [116]	COG	-	Moderate
Li, 2020 [25]	-	BAL; MOV; MOT	Moderate
Miller, 2020 [117]	-	MOV	Moderate
Ismail, 2021 [118]	-	BAL; MOT	Moderate
Okada, 2021 [119]	-	MOT; ADL	Moderate
Zhou, 2021 [120]	QOL; ANX/DEP	BAL; MOV; MOT	Moderate
Ayán Pérez, 2014 [121]	QOL	MOT; MOB	Low
Sharp, 2014 [122]	QOL	BAL; MOV; MOT	Low
Lötzke, 2015 [123]	QOL; FAT	BAL; MOV; MOT	Low
Cassimatis, 2016 [124]	-	ADL	Low
Cusso, 2016 [125]	COG; ANX/DEP; FAT; SLP; APA	-	Low
Yitayeh, 2016 [126]	-	BAL	Low
Mazzarin, 2017 [127]	-	MOB	Low
Connors, 2018 [128]	QOL; COG	BAL; MOV	Low
Carroll, 2020 [129]	-	BAL; MOV; MOB	Low
Morris, 2019 [130]	QOL	BAL; MOB	Low
Pritchard, 2019 [131]	QOL	BAL	Low
Barnish, 2020 [132]	QOL	MOT	Low
Gomes Neto, 2020 [133]	QOL	BAL; MOB	Low
Hidalgo-Agudo, 2020 [134]	-	BAL	Low
Cosentino, 2020 [135]	-	MOV	Low
Oh, 2021 [136]	QOL	BAL; MOT	Low
Hasan, 2022 [137]	COG	BAL; MOV; MOT	Low
Lim, 2005 [138]	-	WLK	Critically low
Crizzle, 2006 [139]	QOL	BAL; STR; ADL	Critically low
Kwakkel, 2007 [140]	-	BAL; MOV	Critically low
Dibble, 2009 [141]	-	BAL	Critically low
De Dreu, 2012 [142]	-	BAL; WLK	Critically low
Foster, 2014 [143]	-	BAL; MOT; MOB	Critically low
Mandelbaum, 2014 [144]	QOL	BAL; MOV; MOT	Critically low
Murray, 2014 [145]	COG	BAL	Critically low
Alves da Rocha, 2015 [146]	QOL	BAL; MOB	Critically low
Shanahan, 2015 [147]	QOL	BAL; MOT; MOV; CARD	Critically low
Aguiar, 2016 [148]	QOL	MOV; MOB; WLK	Critically low
McNeely, 2015 [149]	-	MOV; MOT; MOB; CARD	Critically low
Wu, 2017 [150]	QOL; ANX/DEP	MOT	Critically low
Costa, 2018 [151]	-	MOV; MOB	Critically low
De Freitas, 2020 [152]	-	BAL; MOV	Critically low
Chiong, 2019 [153]	-	BAL; MOV	Critically low
Pupíková, 2019 [154]	-	-	Critically low
Radder, 2020 [155]	QOL	BAL; MOV; MOT	Critically low
Foster, 2021 [156]	-	ADL	Critically low

Colors in the fourth column indicate the level of evidence provided by the systematic review, considering its methodological quality: Green means high level of evidence, yellow means moderate level of evidence, orange means low level of evidence and red means critically low level of evidence.

Abbreviations represent the outcomes improved by physical exercise, as follows: QOF–Quality of life; BRE–Breath; FAT–Fatigue; BAL–Balance; MOV–Movement/walking; MOT–Motor function; FALL–Falls; CARD–cardiorespiratory; MOB–mobility; WLK–walking; COG–cognitive function; ANX/DEP–anxiety/depression; BNDF–brain-derived neurotrophic factor; ADL–activities of daily living; DIS–disease severity; APA–apathy; STR–strength; HUM–humor; SLP–sleep; WLB–well-being; FLX–flexibility.

## Discussion

An expressive amount of physical exercise intervention protocols focusing on the population with PD has been proposed, assessed and synthesized in scientific literature. This umbrella review found one hundred and thirty-nine SR dedicated to synthesizing the evidence from clinical trials (Fig 1). It also pointed out the outcomes improved by the practice of each category of exercise and determined, based on AMSTAR 2, the level of evidence available to recommend such protocols to people with PD (Table 4). High quality evidence revealed that movement, walking, mobility, motor function and equilibrium benefit from aerobic exercises, while strength exercises improve strength, walking and cardiorespiratory function. Also, it was found that combined exercises improve sleep and the quality of life, and the sensory-motor activities improve balance and mobility. Finally, other protocols, such as aquatic exercises, music-based and dance exercises improve motor function, balance, mobility and prevent falls of people with PD (Table 4).

Still, a low number of high-quality studies provided evidence for such conclusions (Table 4). The methodological quality of the systematic reviews, as assessed by AMSTAR 2 [18], was classified as low or critically low in 63% of the cases. Only 64% of the reviews provided quantitative measures of effect across interventions via meta-analysis. A well-conducted, quality meta-analysis relies on consistent primary data and is supported by consistent statistical inferences, improving the decision-making process toward the most effective intervention [157]. Besides, the lack of a priori protocol registration, lack of justification for exclusion of individual studies and possible publication bias were identified as methodological drawbacks (S3 Table). The method for the conduct of systematic reviews has undergone changes and improvements that should be extensively acknowledged by authors and journal revisors aiding at the publication of high standard reviews that aid robust evidence [158].

The pooled number of participants with PD was 54,501, including populations from all the continents. The lowest number of participants included in a systematic review was 37, while the highest was 7,998 (Table 3). Sex of the participants was informed by 65% of the studies, with the presence of both sexes. Males have been shown with a higher risk of developing PD compared to females [1]. Also, age ranged from 18 to 90 years, with 91% of the participants older than 50 years. PD is age-related, with prevalence peaking in 85-89-years range. Moreover, its lethality has been shown to raise with age [1]. The Global Burden of Diseases revealed that age-standardized prevalence of PD in 2016 maintained the 1990 pattern, with men presenting a 1.40 (95% uncertainty interval 1.36–1.43) times higher prevalence than women [1].

Most studies did not report the time elapsed since the onset of PD (Table 3). For the studies that reported, a variation of 0.3 to 17 years was observed. Characteristically, the progression of PD in terms of symptoms and severity may oscillate [159]. The staging of the functional disability caused by PD may be assessed and monitored using the 5-point Hoehn & Yahr scale [4]. The level of functional compromise was expressed by 82% of the studies and varied from 1 (unilateral involvement only) to 4 (severe disability; still able to walk or stand unassisted) (Table 3).

Motor outcomes were assessed in 91% of the studies. Balance was the most studied motor outcome and was determined using the Berg Balance Scale (BBS) and the Activities-Specific Balance Confidence Scale (ABC). The BBS scale measures balance skills in sitting, standing and changing positions, while the ABC scale assesses balance confidence and fear of falling that may be protective for dangerous activities [160]. Non-motor outcomes were assessed in 68% of the studies. Quality of life was the most assessed non-motor outcome (92%), mainly with the Parkinson´s Disease Questionnaire (PDQ-39). Thirty-nine items address difficulties facing eight domains of daily living, which involve mobility, activities of daily living (ADL) emotional well-being, stigma, social support, cognition, communication and physical discomfort [161].

### Aerobic exercises

Twenty-four different interventions were categorized as aerobic exercises in the included systematic reviews (Table 2). High-quality studies revealed benefit on balance, movement/walking, motor function, mobility, walking and endurance/cardiorespiratory function by aerobic exercises [21,22,23] (Table 4). Aerobic exercises also have been shown to reduce cardiovascular diseases, to improve bone health and to lower mortality rates in people with PD [162]. Additionally, they improve VO_2max_ performance and attenuate motor symptoms without the use of medication. No high-quality systematic review evidenced benefit of non-motor outcomes by aerobic exercises (Table 4). Even so, moderate quality studies found improvement in quality of life after performing aerobic exercise protocols [25,26]. Protocol registration prior to commencement of the review and adequate literature search were the critical domains that lowered rating of these studies. Exercise dosage, expressed by frequency, volume, intensity and duration of the protocol, among other factors, may be better reported in some of the critically low- and low-quality systematic reviews. Optimal exercise dosage remains a challenge for specialized professionals. Although high-intensity exercises are known as more effective than moderate-intensity exercises, evidence on dose-response relationship for people with PD is still required [162]. Even so, aerobic exercise has been shown to reduce long-term prodromal, early and intermediate stages of motor and non-motor symptoms in people with PD [159]. Therefore, reduction of tremor and bradykinesia, improvement in balance and gait, and improved quality of life are expected.

### Strength exercises

Strength exercises improved quality of life in almost half of the studies (Table 4). Still, only one moderate-quality study addressed this outcome. Also, the only high-quality study and the other moderate-quality studies addressed walking, strength, endurance/cardiorespiratory function, motor function, mobility and balance, showing improved results. Lima et al. [44] found positive effects of strength exercises on walking and strength, but not on motor function. The authors recommended strength training to improve walking. The methodological ranking was moderate due to lack of protocol registration, justification for exclusion of primary studies and assessment of probable risk of publication bias. Saltychev et al. [43] was ranked with high evidence and found improvement of walking, muscle strength and cardiorespiratory endurance by strength training.

### Combined exercises

Combined exercises are planned to impact more than one component of physical fitness, for instance, cardiorespiratory capacity, flexibility and strength, simultaneously [20]. Thirty-six interventions were categorized as combined exercises, involving Pilates, different types of dances, Yoga and cycling. Two high-quality studies observed improvement of non-motor outcomes, namely sleep [57] and quality of life [56]. Four moderate-quality studies showed improved cognitive function, breathing capacity and the presence of the Brain-Derived Neurotrophic Factor (BDNF), which is a modulator of neurodevelopment and neuroprotection [163]. Blood levels of BDNF decrease in people with PD [164], and the recovery of BDNF blood levels by combined exercises suggest possible non-pharmacological interventions to enhance neuroprotection. Muscle strength, cardiorespiratory capacity, balance, motor function, movement and walking were improved by combined exercises, as revealed by moderate-quality studies (Table 4). These studies were rated down due to one to two critical domains, including lack of protocol registration prior to commencement of the review, justification for excluding individual studies and assessment of likely impact of publication bias.

### Sensory-motor activities

Sensory-motor activities promote emotional, mental, behavioral, social and spiritual interactions that affect one´s health [20]. This category involved ten types of exercises, including Tai Chi, virtual reality, exergames, Yoga and Pilates. Almost all studies of sensory-motor activities addressed some motor outcome. Quality of life and anxiety/depression were the non-motor outcomes analyzed. One high-quality study [74] revealed improvement of balance and mobility with sensory-motor activities. Besides, most moderate-quality studies observed positive effect of the exercise on motor function, fall prevention, mobility, walking, flexibility and, mainly, on balance (Table 4). Moderate quality rating was due to lack of protocol registration, adequacy of literature search and to lack of assessment of likely impact of publication bias.

### Other protocols

A variety of exercise protocols were otherwise classified. The eighty-four types of exercises categorized as ’other protocols’ did not present sufficient data regarding the protocols such as volume and/or intensity, a fact that limits the analyses related to the exercise dose and the observed effect. Still, two high-quality studies revealed that water-based studies had a positive effect on quality of life, balance, fall prevention and mobility [105,106]. Also, that functional task training improved motor function [104] and that a combination of physical therapy, resistance, treadmill and strategy training, dance, aerobic exercise, balance and gait training and Tai Chi improved fall prevention [107]. Moderate quality studies, which may present more than one flawed non-critical domain [152] revealed benefits of cognition by dance-based exercises, such as Tango, Irish dance, Jazz and Ballet [115,116]. Different exercises, such as aerobic, force, balance Qigong and other exercises, and music-based movement improved quality of life [108,120]. Moderate-quality evidence of benefit of balance as the most assessed motor outcome was found by the several different exercises [25,108–112,114,118,120]. Mobility, movement/walking, fall prevention, mobility, walking, activities of daily living and motor function also improved with the different types of exercise proposed (Table 4).

Evidence on safety, adherence and maintenance of interventions based on physical exercise programs for patients with PD must be constantly reviewed. Concerns with the practice of physical exercises by people with PD involve musculoskeletal injuries and cardiovascular risks, which requires an individual prescription considering the possibilities and limitations of each patient [36,165].

Considering the studies analyzed in this umbrella review, it is not possible to indicate the best intervention protocol, mainly due to the diversity of physical exercise protocols and the characteristics of the patients included in the SR. It should also be noted that only 45% of the SR reported adverse effects related to the interventions (pain, injuries and worsening of general health conditions and comorbidities).

Even so, evidence suggests that exercise is a promising, economical, low-risk intervention, which improves motor and non-motor symptoms and may be prescribed and encouraged for people with PD. Based on that, research on the therapeutic efficacy of exercise is a growing and exciting area [166].

### Implications for clinical practice

Health professionals may recommend physical exercises to people with PD, considering the specific characteristics and individualities of each one.

Any type of exercise may be indicated to improve balance and mobility. Aerobic exercises would be indicated to improve balance, movement, motor function, mobility, walking, cardiorespiratory fitness and BDNF. Strength exercises can improve strength and quality of life, while protocols that combine different types of exercises can improve quality of life, cognitive function, sleep, balance, movement, motor function, mobility, walking, cardiorespiratory fitness, strength, and BDNF.

Sensorimotor activities may be indicated to improve balance, motor function, mobility, walking and to reduce falls, the latter being the only intervention with positive results for this population. Various physical activities such as those performed in the aquatic environment or dancing are indicated to improve quality of life, cognitive function, sleep, balance, movement, mobility, motor function, walking and physical activities of specific tasks improve responses in activities of daily living.

### Implications for research

The number of published and analyzed studies shows that the issue has been widely studied, due to its relevance and potential impact on the lives of patients with PD. On the other hand, a considerable part of the analyzed studies has important limitations that compromise the production of consistent scientific evidence to support professionals who work with the prescription of physical exercises for patients with PD.

The findings of this study reveal the need for greater methodological care in conducting RCTs, especially in relation to instruments for data collection and types of outcomes analyzed, description of exercise protocols and adverse effects related to interventions.

## Conclusion

In conclusion, evidence suggests that physical exercise improves the motor outcomes of balance, mobility, movement, motor function and walking. Combined exercises may have positive effects on motor and non-motor outcomes. Exercise in PD has been studied because of its relevance and potential impact. However, the evidence-based decision process is hindered by the limited methodological quality of most reviews.

This study presents information and directions to produce evidence with better methodological quality in RCTs and RS for the effects of physical exercise to people with PD, aiding at formulation of policies and guidelines.

## Supporting information

S1 Table Electronic search strategies. (DOCX)

S2 Table Characteristics on the included studies, participants, interventions and outcomes. (DOCX)

S3 Table AMSTAR 2 assessment of the included systematic reviews. (DOCX)

S4 Table PRISMA checklist–Scoping (umbrella) review. (DOCX)
