# Supplementary material for: Physical exercise and its effects on people with Parkinson’s disease: Umbrella review
Source: PLoS One. 2023 Nov 2;18(11):e0293826. doi: 10.1371/journal.pone.0293826 (PMC10621990; doi:10.1371/journal.pone.0293826)
Supplement: S1 Table — (DOCX) [file pone.0293826.s001.docx]

**S1 Table. Electronic search strategies**

| **Cochrane Library** | ("Parkinson's Disease" OR "Idiopathic Parkinson's Disease" OR "Lewy Body Parkinson Disease"  OR "Lewy Body Parkinson's Disease"  OR "Primary Parkinsonism"  OR "Parkinsonism, Primary"  OR "Parkinson Disease, Idiopathic"  OR "Parkinson's Disease"  OR "Parkinson's Disease, Idiopathic"  OR "Parkinson's Disease, Lewy Body"  OR "Idiopathic Parkinson Disease"  OR "Paralysis Agitans") AND (Exercise* OR "Physical Activity" OR "Activities, Physical" OR "Activity, Physical" OR "Physical Activities" OR "Exercise, Physical" OR "Exercises, Physical" OR "Physical Exercise" OR "Physical Exercises" OR "Acute Exercise" OR "Acute Exercises" OR "Exercise, Acute" OR "Exercises, Acute" OR "Exercise, Isometric" OR "Exercises, Isometric" OR "Isometric Exercises" OR "Isometric Exercise" OR "Exercise, Aerobic" OR "Aerobic Exercise" OR "Aerobic Exercises" OR "Exercises, Aerobic" OR "Exercise Training" OR "Exercise Trainings" OR "Training, Exercise" OR "Trainings, Exercise") AND ("systematic review" OR "systematic literature review" OR "systematic scoping review" OR "systematic narrative review" OR "systematic qualitative review" OR "systematic evidence review" OR "systematic meta-review" OR "systematic critical review" OR "systematic mixed studies review" OR "systematic mapping review" OR "systematic cochrane review" OR "systematic search and review" OR "systematic integrative review" OR "scoping review") |
| --- | --- |
| **CINAHL Plus via EBSCOhost** | ("Parkinson's Disease" OR "Idiopathic Parkinson's Disease" OR "Lewy Body Parkinson Disease"  OR "Lewy Body Parkinson's Disease"  OR "Primary Parkinsonism"  OR "Parkinsonism, Primary"  OR "Parkinson Disease, Idiopathic"  OR "Parkinson's Disease"  OR "Parkinson's Disease, Idiopathic"  OR "Parkinson's Disease, Lewy Body"  OR "Idiopathic Parkinson Disease"  OR "Paralysis Agitans") AND (Exercise* OR "Physical Activity" OR "Activities, Physical" OR "Activity, Physical" OR "Physical Activities" OR "Exercise, Physical" OR "Exercises, Physical" OR "Physical Exercise" OR "Physical Exercises" OR "Acute Exercise" OR "Acute Exercises" OR "Exercise, Acute" OR "Exercises, Acute" OR "Exercise, Isometric" OR "Exercises, Isometric" OR "Isometric Exercises" OR "Isometric Exercise" OR "Exercise, Aerobic" OR "Aerobic Exercise" OR "Aerobic Exercises" OR "Exercises, Aerobic" OR "Exercise Training" OR "Exercise Trainings" OR "Training, Exercise" OR "Trainings, Exercise") AND ("systematic review" OR "systematic literature review" OR "systematic scoping review" OR "systematic narrative review" OR "systematic qualitative review" OR "systematic evidence review" OR "systematic meta-review" OR "systematic critical review" OR "systematic mixed studies review" OR "systematic mapping review" OR "systematic cochrane review" OR "systematic search and review" OR "systematic integrative review" OR "scoping review") |
| **EMBASE** | ('parkinson disease'/exp OR 'parkinson disease' OR 'parkinson`s disease' OR 'parkinsons disease' OR 'paralysis agitans' OR 'parkinson dementia complex' OR 'parkinson disease, postencephalitic' OR 'parkinson disease, secondary' OR 'parkinson disease, symptomatic' OR 'postencephalitic parkinson disease' OR 'secondary parkinson disease' OR 'symptomatic parkinson disease' OR 'idiopathic parkinson disease'/exp) AND ('exercise'/exp OR 'effort' OR 'exercise' OR 'exercise training' OR 'physical conditioning, human' OR 'physical effort' OR 'physical exercise' OR 'physical exertion' OR 'physical activity'/exp OR 'activity, physical' OR 'physical activity' OR 'acute exercise'/exp OR 'isometric exercise'/exp OR 'exercise, isometric' OR 'isometric endurance' OR 'isometric endurance test' OR 'isometric exercise' OR 'isometric training' OR 'aerobic exercise'/exp OR 'aerobics exercise' OR 'exercise, aerobic' OR 'training'/exp) AND ('systematic review'/exp OR 'review, systematic' OR 'systematic review' OR 'literature'/exp OR 'review literature' OR 'review literature as topic') |
| **PEDro** | #1 Parkinson’s Disease AND Exercis*  #2 Systematic Review  #1 AND #2  Abstract & Title: parkinson treadmill  Therapy: fitness training  Subdiscipline: neurology  Method: clinical trial  (Search terms matched with AND) |
| **MEDLINE via PubMed:** | ("Parkinson's Disease" OR "Idiopathic Parkinson's Disease" OR "Lewy Body Parkinson Disease"  OR "Lewy Body Parkinson's Disease"  OR "Primary Parkinsonism"  OR "Parkinsonism, Primary"  OR "Parkinson Disease, Idiopathic"  OR "Parkinson's Disease"  OR "Parkinson's Disease, Idiopathic"  OR "Parkinson's Disease, Lewy Body"  OR "Idiopathic Parkinson Disease"  OR "Paralysis Agitans") AND (Exercise* OR "Physical Activity" OR "Activities, Physical" OR "Activity, Physical" OR "Physical Activities" OR "Exercise, Physical" OR "Exercises, Physical" OR "Physical Exercise" OR "Physical Exercises" OR "Acute Exercise" OR "Acute Exercises" OR "Exercise, Acute" OR "Exercises, Acute" OR "Exercise, Isometric" OR "Exercises, Isometric" OR "Isometric Exercises" OR "Isometric Exercise" OR "Exercise, Aerobic" OR "Aerobic Exercise" OR "Aerobic Exercises" OR "Exercises, Aerobic" OR "Exercise Training" OR "Exercise Trainings" OR "Training, Exercise" OR "Trainings, Exercise") AND ("systematic review" OR "systematic literature review" OR "systematic scoping review" OR "systematic narrative review" OR "systematic qualitative review" OR "systematic evidence review" OR "systematic meta-review" OR "systematic critical review" OR "systematic mixed studies review" OR "systematic mapping review" OR "systematic cochrane review" OR "systematic search and review" OR "systematic integrative review" OR "scoping review") |
| **LILACS** | ("Parkinson's Disease" OR "Idiopathic Parkinson's Disease" OR "Lewy Body Parkinson Disease"  OR "Lewy Body Parkinson's Disease"  OR "Primary Parkinsonism"  OR "Parkinsonism, Primary"  OR "Parkinson Disease, Idiopathic"  OR "Parkinson's Disease"  OR "Parkinson's Disease, Idiopathic"  OR "Parkinson's Disease, Lewy Body"  OR "Idiopathic Parkinson Disease"  OR "Paralysis Agitans") AND (Exercise* OR "Physical Activity" OR "Activities, Physical" OR "Activity, Physical" OR "Physical Activities" OR "Exercise, Physical" OR "Exercises, Physical" OR "Physical Exercise" OR "Physical Exercises" OR "Acute Exercise" OR "Acute Exercises" OR "Exercise, Acute" OR "Exercises, Acute" OR "Exercise, Isometric" OR "Exercises, Isometric" OR "Isometric Exercises" OR "Isometric Exercise" OR "Exercise, Aerobic" OR "Aerobic Exercise" OR "Aerobic Exercises" OR "Exercises, Aerobic" OR "Exercise Training" OR "Exercise Trainings" OR "Training, Exercise" OR "Trainings, Exercise") AND ("systematic review" OR "systematic literature review" OR "systematic scoping review" OR "systematic narrative review" OR "systematic qualitative review" OR "systematic evidence review" OR "systematic meta-review" OR "systematic critical review" OR "systematic mixed studies review" OR "systematic mapping review" OR "systematic cochrane review" OR "systematic search and review" OR "systematic integrative review" OR "scoping review") |
| **SPORTDiscus via EBSCOhost** | ("Parkinson's Disease" OR "Idiopathic Parkinson's Disease" OR "Lewy Body Parkinson Disease"  OR "Lewy Body Parkinson's Disease"  OR "Primary Parkinsonism"  OR "Parkinsonism, Primary"  OR "Parkinson Disease, Idiopathic"  OR "Parkinson's Disease"  OR "Parkinson's Disease, Idiopathic"  OR "Parkinson's Disease, Lewy Body"  OR "Idiopathic Parkinson Disease"  OR "Paralysis Agitans") AND (Exercise* OR "Physical Activity" OR "Activities, Physical" OR "Activity, Physical" OR "Physical Activities" OR "Exercise, Physical" OR "Exercises, Physical" OR "Physical Exercise" OR "Physical Exercises" OR "Acute Exercise" OR "Acute Exercises" OR "Exercise, Acute" OR "Exercises, Acute" OR "Exercise, Isometric" OR "Exercises, Isometric" OR "Isometric Exercises" OR "Isometric Exercise" OR "Exercise, Aerobic" OR "Aerobic Exercise" OR "Aerobic Exercises" OR "Exercises, Aerobic" OR "Exercise Training" OR "Exercise Trainings" OR "Training, Exercise" OR "Trainings, Exercise") AND ("systematic review" OR "systematic literature review" OR "systematic scoping review" OR "systematic narrative review" OR "systematic qualitative review" OR "systematic evidence review" OR "systematic meta-review" OR "systematic critical review" OR "systematic mixed studies review" OR "systematic mapping review" OR "systematic cochrane review" OR "systematic search and review" OR "systematic integrative review" OR "scoping review") |
| **Science Direct (Elsevier)** | "Parkinson's Disease" AND (Exercise OR "Physical Activity") AND ("systematic review" OR "systematic literature review" OR "systematic scoping review") |
| **Scopus (Elsevier)** | **TITLE=ABS=KEY** "Parkinson's Disease" AND (Exercise OR "Physical Activity") AND ("systematic review" OR "systematic literature review" OR "systematic scoping review" |
