# Supplementary material for: Physical exercise and its effects on people with Parkinson’s disease: Umbrella review
Source: PLoS One. 2023 Nov 2;18(11):e0293826. doi: 10.1371/journal.pone.0293826 (PMC10621990; doi:10.1371/journal.pone.0293826)
Supplement: S2 Table — (DOCX) [file pone.0293826.s002.docx]

**S2 Table. Characteristics of participants, interventions and outcomes**

| **PARTICIPANTS** | | | | | **INTERVENTIONS** | | | | | | **OUTOCOMES** |
| --- | --- | --- | --- | --- | --- | --- | --- | --- | --- | --- | --- |
| **First author, year** | **Studies/**  **participants** | **Age range** | **Parkinson´s level (H & Y)** | **Duration range** | **Type** | **Session time (min)** | **Duration (weeks)** | **Frequency (days)** | **Intensity** | **Control** | **Motor/non-motor** |
| AEROBIC EXERCISE | | | | | | | | | | | |
| Herman, 2009 [34] | 14/298 | - | - | - | Aerobic exercise, strength training, rhythm retraining, body weight support, motor learning, corticomotor output ability | 20-60 | 4-12 | 3-4 | Low/High | Maintain group activity level | QOL; FAT; BAL; MOV; MOT; FALL; CARD |
| Jambeau, 2011 [35] | 13/303 | 61-79 | 1-4 | 1.5-17 | Treadmill march | 10-90 | 1-12 | 2-7 | 50-80% max speed; 70-80% CF; 0.5-4 km/h | - | QOL; BAL; MOT; MOB; WLK |
| Shu, 2014 [28] | 18/901 | 62-72 | 1-4 | 6.4 | Aerobic exercise: treadmill training, Tai Chi, walking, dancing | 20-70 | 3-24 | - | 80% intense aerobic exercise | Usual care, stretching, resistance exercises, physical therapy and other exercises | QOL; BAL; MOV; MOT |
| Lamotte, 2015 [36] | 8/1210 | - | 1-3 | - | Endurance: High intensity interval training (HIIT)-treadmill, low-treadmill, stretching, resistance training, flexibility, balance, cycling, elliptic, running machine, HIIT, Bodyweight strength training (BWST), low intensity | 20-60 | 8-24 | 2-3(5-7) | HIIT=60-85% HR; low=50-65% CFmax | Education, usual care, home base, no intervention | QOL; COG; ANX/DEP; FAT; BAL; MOV; MOT; MOB; CARD |
| Mehrholz, 2015 [15] | 18/633 | 58-74 | 1-3 | 1-13 | Treadmill training | 20-60 | 4-24 | 3-5 | 40-80% speed | - | MOV; WLK |
| Cascaes da Silva, 2016 [29] | 5/195 | 62-72.7 | 1.6-2.8 | 3.7-7.8 | Nordic walking | 60-70 | 6-24 | - | 8-90% of CF; PSE-BORG | Flexibility and relaxation | QOL; BAL; MOV; MOT; MOB |
| Bombieri, 2017 [37] | 9/146 | - | 1-4 | - | Nordic walking | 60-70 | 6-24 | 2-3 | High intensity | LSVT®BIG, unsupervised home exercise, walking or flexibility, relaxation training | QOL; MOV; MOB; WLK |
| Flach, 2017 [38] | 8/289 | - | - | - | Aerobic exercise | 30-60 | 4-24 | 3-5 | 60-75% CFmax.  70-80% CFreserves; PSE-  BORG 11-15/20 | Stretching and resistance training, light exercise only, no exercise | ANX/DEP |
| Mackay, 2017 [24] | 3/44 | 39-77 | - | - | Treadmill and bicycle ergometer, force and  relaxation, exercise bike and interval training | 40-60 | 4-8 | 3-5 | <60% CF | No exercise | BDNF |
| Alwardat, 2018 [30] | 7/286 | 67.4-74.6 | 2-3 | - | Robot-assisted gait training (RAGT) | 30-45 | 4 | 3-5 | 1-3 km/h | Treadmill, conventional physical therapy, training | BAL; MOV; FALL; MOB |
| Robinson, 2019 [22] | 11/518 | 58.2-72.5 | 1-3 | 4-16.2 | Sit down to stand up, walking, running machine, functional exercise, nordic walking, walking BWSTT, task specific, exercise at home, circuit training | 20-60 | 4-12 | 2 | 50-85 CF | No intervention | MOV; MOB; WLK |
| Seuthe, 2019 [39] | 7/118 | 58.3-66.8 | 2.1-2.6 | 5.4-14.6 | Split belt treadmill (SBT) | 2-20 | - | - | 1.8-4 km/h | Healthy control, walking protocol | MOV; WLK |
| Aburub, 2020 [40] | 9/279 | 56.8-70.2 | 1-3 | - | Cycling, treadmill, walking, Qigong, cross training, rowing ergometer, balance, flexibility | 20 | 6-24;  1 study=68 | 2-5 | 40-85% CF | Usual activities | ADL |
| De Santis, 2020 [31] | 13/318 | 50-70 | 1-3 | 1.5-8 | Nordic walking | 60-70 | 6-12  1 study= 24  1 study = 1  1 study= 4 | 2-3  1 study=5 | - | Free walking without poles, BIG structured physical therapy program, unsupervised home exercises, flexibility and relaxation training, standard doctor | QOL; COG; ANX/DEP; FAT; DIS; APA; BAL; MOB; WLK |
| Li, 2020 [25] | 9/444 | 59.3-68.1 | 1-3 | 0.3-8.5 | Treadmill/Nordic, bicycle, run | 20-90 | 8-24 | 2-4(5-7) | 50-80%  Freserve | Routine exercises, exergames, exercises at home | QOL; BAL; MOV; MOT; STR |
| Miner, 2020 [41] | 6/139 | 60.6-69.9 | 1-3 | 3.4-7 | Forced exercise cycling | 40-60 | 8-12 | 2-5 | PSE-BORG; 50-80% CF | Static cycling, stretching or no intervention | MOT; MOB |
| Rodriguez, 2020 [32] | 7/419 | 58-69.8 | 1-3 | - | Vigorous-intensity exercise-aerobic (VIE), alone or in combination | 10-60 | 8-26 | 3-4 | PSE-BORG (15-17); 70-85% CFmax. 60% CFreserves; 64% VO^2^max | Light exercise only: stretching, resistance training, moderate exercise, flexibility, in the exercise, calisthenics | QOL; HUM; BAL; MOT; WLK; STR; CARD |
| Braz de Oliveira, 2021 [42] | 10/411 | 30-80 | 1-3 | 4.8-8.9 | Treadmill, walking | 20-70 | 4-26 | 2-4 | 60-85%CF | Usual care, medication, and physical activity “passive controls”, and “active controls” | QOL; MOT; MOB; MOV; STR |
| Lorenzo Garcia, 2021 [23] | 12/552 | 57-71 | 1-4 | 1.2-11.4 | Bodyweight support (BWS), gait training or robotic systems with BWS | 30-45 | 4-8 | 3-5 | 0.5-3 km/h | Conventional physical therapy training +  Occupational therapy): balance or without intervention | BAL; MOV; MOT; WLK |
| Tiihone, 2021 [26] | 22/505 | 59-71 | 1.5-2.6 | 3.3-10.5 | Cycling | 30-75 | 1-12 | 1-5 | 50-80%CF | NR | BAL; MOT; MOV; MOB; WLK; QOL |
| De Almeida, 2022 [27] | 27/1152 | 57.6-72.2 | 1-4 | 0.4-17.9 | Cycloergometer, treadmill and (BWSTT), walking, Nordic walking, aerobic physical therapy | 30-80 | 4-64 | 2-5 | 50-80%CF  40-80%VO^2^ | Treadmill, walking, physical therapy, no intervention, education | MOT |
| Salse-Batán, 2022 [33] | 12/998 | 60.3-71.2 | 1-4 | 5.0-18.1 | Nordic walking | 35-70 | 4-24 | 2-5 | 60-80%CF | Walking, training at home, no intervention | FAT; ANX/DEP; QOL; BAL; MOV; MOB; WLK; CARD |
| RESISTANCE EXERCISE | | | | | | | | | | | |
| Lima, 2013 [44] | 4/92 | 57-75.7 | 1.8-2.5 | - | PRE; Balance, treadmill, cycle ergometer | 30-60 | 8-24 | 2-3 | PSE-BORG 13-15; 60-80%1MR | Usual care | MOT; WLK; STR |
| Brienesse, 2013 [47] | 5/288 | 50-82 | - | 1.1-12.3 | RT: full body resistance training | 45-60 | 8-12 | 1-3 | (60-70% 1MR) PSE-BORG 7-13 | Standard care, calisthenics, stretching, upper and lower limb resistance training, full body resistance training | MOT; MOB; STR; CARD |
| Chung, 2016 [48] | 7/401 | 58.6-70.8 | 1.8-3 | 5.9-11.1 | Resistance training intensity: machine, body weight | - | 8-24  1 study=104 | 2-3 | 30-80 (MR) | Exercise, Tai Chi, treadmill exercise, stretching, no intervention | QOL; BAL; MOT; STR |
| Cruickshank, 2015 [45] | 8/1210 | 59-68.7 | 1-5 | - | Strength training: full body or lower | - | 8-12  1 study=104 | 2-3 | 30-80 (MR) | Activities of daily living, standard care, Low and high intensity treadmill | QOL; HUM; BAL; MOT; MOB; WLK; STR |
| Roeder, 2015 [49] | 9/425 | 67.7 | 1-4 | 7.1 | Strength training program | 40-60 | 6-24 | 2-3 | %MR; PSE-BORG, Until failure | Control group with or without interventions | STR |
| Tillma, 2015 [50] | 7/172 | 59.1-73.2 | 1-3 | 6.2-8.7 | Progressive resistance training | 40-60 | 8-24 | 1-3 | 60%MR; 50-75% PSE-BORG; 4CFmax | Standard DP exercise management, running machine | QOL; BAL; MOV; WLK; STR; CARD; FLX |
| Saltychev, 2016 [43] | 12/383 | 59-71 | - | - | Progressive resistance training | 40-90 | 6-24 (104) | 2-3 | - | Usual care, usual activity, strengthening, endurance and balance exercises, standard care, running machine, placebo | QOL; COG; HUM; FAT; MOV; MOB; WLK; STR; CARD |
| Ramazzina, 2017 [51] | 13/458 | 58.6-75.7 | 1.5-3 | - | Strength training | 15-60 | 1-12(104) | 1-5 | PSE-BORG (11-14); 30-80%MR | Physiotherapy, treadmill training, and balance training | QOL; BAL; MOV; FALL; STR; CARD; FLX |
| Rodríguez, 2020 [53] | 5/111 | 59.4-70.4 | 1-4 | - | IMT and EMT | 30 | 4-12 | 5-6 | 15-60%MI;  50-75%ME | Sham (EMT) 5 × 5 repetitions, placebo, another rehabilitation program | QOL; STR; BRE |
| Li, 2020 [46] | 31/1239 | 58.8-79.5 | 1-4 | 4.1-10.6 | Lower member RT | 15-90 | 6-104 | 1-3 | Low, high | Balance training, stretching, no intervention, treadmill exercise, sham exercise | QOL; BAL; MOV; STR |
| Van der Wetering-van Dongen, 2020 [52] | 10/209 | 58.8-70.5 | 1- 4 | - | IMST: inspiratory muscle strength training, EMST, expiratory muscle strength training | 30 | 4-12 | 3-6 | 50-75%MI/ME | H^2^O protocol, MIP/MEP residential base | QOL; STR; BRE |
| Braz de Oliveira, 2021 [54] | 10/270 | 45-90 | 1-3 | 6-11.1 | Endurance (strength) training | 30-70 | 8-24 | 1-2 | 40-80%MR | Physiotherapy, usual activities, medication, education | BAL; MOV; MOB; CARD; STR; FLX; QOL |
| de Lima, 2022 [55] | 10/556 | - | - | - | Endurance (strength) training | 30-45 | 8-12 | NR | - | NR | BAL; MOV; MOT; STR; QOL |
| COMBINED EXERCISE | | | | | | | | | | | |
| Tambosco, 2014 [70] | 36/505 | 61-74 | 1- 4 | - | Strength training | 40 | 10-68 | 3 | 50-80%Freserve | Ergonometers | QOL; COG; ANX/DEP; BAL; MOV; MOT; FALL; STR; CARD; ADL |
| Uhrbrand, 2015 [58] | 15/793 | 59-68 | 1- 3 | 2.9-8.8 | RT; ET; Other intensive training modalities | 20-60 | 4-12  1 study =68  1 study  =104 | 1-3 | 40-50%; 70-80% CFreserve;60-75% CFmax. <5 PSE-BORG | Usual activities, flexibility and strengthening, resistance, step training with tips | QOL; BAL; MOT; WLK; STR; CARD |
| Cascaes da Silva, 2016 [29] | 14/1205 | 44-70.2 | 1.5-2.5 | 3.9-9 | RT, Movement Strategy Group, dance, EIP (strength and aerobic training, FBF - flexibility-balance and functional training, home-based, Tai Chi, Physiotherapy, High-intensity physical training, PF - ParkFit program | 40-60 | 6-64 | 2-3 | 30–80%MR; 80% average speed; 60–85% HR; PSE-BORG 7–13) | Exercise group, standard care, standard physiotherapy, non-intervention, stretching and resistance | QOL |
| Reynolds, 2016 [71] | 13/581 | 59.5-71 | - | - | Aerobic and resistance exercise | 20-90 | 8-48 | 2-4 | Low or high intensity | Usual care, (LSVTBIG), stretching and resistance | COG; HUM; SLP |
| da Silva, 2018 [66] | 9/253 | 59-71 | 1-3 | 4.7-11.2 | Treadmill training, Argentine tango, exercise bike, Tai Chi, WiiFit™ Exercise, strength exercises, cycling, rowing or treadmill, Multimodal exercises (flexibility, stretching, muscular resistance | 45-90 | 4-24 | 2-3 | 1-2km/h; 80%VO^2^max.; 75%CFmax. PSE-BORG; 60±85%CFmax. 5±10%MR increment per week | Physical exercises, Tai-Chi, Latin dance, exercises with resistance bands, motor coordination, stretching, strengthening and axial mobility, usual routine | COG |
| Hirsch, 2018 [67] | 35/79 | 68* | 1-3 | 6.8-11.8 | Stationary cycling, running machine, resistance training, Wii Fit Balance board system, multimodal exercise | 45-60 | 4-12 | 3-5 | 60-80% CFreserves, 60-90 RPM, 3.5-4 km/h | NR | BDNF; MOT |
| Ni, 2018 [59] | 40/1656 | - | 1-4 | - | LSVT BIG Program, multidimensional physical training, treadmill and cycling training, resistance training, Tai Chi, Yoga, dance and boxing, importance of task-specific training | 20-60 | 4-12 | 1-3(7) | PSE-BORG, MR, CF, Total hours | NR | BAL; MOV; WLK |
| Stuckenschneider, 2019 [60] | 35/113 | 62.1(68*)75.9 | 1-4 | 4.1-11.6 | AE = aerobic exercise, RE = resistance exercise, EC = coordination exercise, Virtual reality augmented balance training | 20-60 | 4-16  1 study =17  1 study  =26 | 1-3 | 50-75%CFmax. 60-80%CFmax. 40-50%CFreserve and 60-70% CFreserve | Usual care, psychosocial | COG |
| Chen, 2020 [56] | 20/1143 | 61.8-73 | - | 2.9-8 | Strengthening and balance exercises, walking, treadmill walking, RT, stretching strengthening exercise, Tai chi, exercises, functional training, gait training, balance training, Foxtrot, Power Yoga, Aerobic sessions | 30-120 | 3-25 | 1-5  1 study  =7 | - | No intervention | QOL |
| Choi, 2020 [61] | 18/1144 | - | 1-5 | 3.6-11.2 | Walking training, strength or flexibility training, balance training, aerobic training, agility, virtual reality training, tango, breathing training, supported body weight | 30-120 | 4-56 | 2-6  1 study  =1 | %CF, %MR, Speed | Conventional physiotherapy | BAL; MOV; MOT; WLK |
| Costa, 2020 [68] | 11/925 | 30-80 | 1.9-2.3 | - | Balance, resistance training, Strength training, Yoga, aerobic training, Pilates, running machine | 40-70 | 8-24 | 1-4 | Moderate-intense | No intervention | QOL; DIS; BAL; MOT |
| Johansson, 2020 [62] | 13/210 | 53.5-71.6 | 1-2 | - | Motor rehabilitation, multidisciplinary, bicycle, dance, treadmill, HIIT, BWS, flexibility, relaxation, coordination, posture and walking, (2) treadmill (aerobics) and Wii Fit balance board, (3) motor therapy, strength and balance | 40-60 | 4-12  (1 study; 1 week) | 2-5  1 study  =1  3 studies  =15 | 60-80%CF | Bicycle, running machine, calistenic, no intervention | BDNF |
| McMahon, 2020 [63] | 12/371 | 51-70.3 | 1-3 |  | Respiratory muscle strength training, aerobic/endurance/  neurodynamic, aerobic exercise and Qigong, Iyengar Yoga, exergaming and functional training, cycling and functional training, and general strength training,  functional | 60 | 6-16 | 5-6 | 50-75%ME-MI | IMT-low load, no resistance, threshold PEP, crossover, EMST-SHAM | WLK; BRE |
| Smith, 2020 [72] | 9/387 | 48-89 | 1-3 | 5.4-9.2 | Running machine, dance, flexibility, Wi Fit | 15-60 | 8-13/104 | 2-3 | 70-70%CF | No intervention | MOV; MOB |
| Cristini, 2021 [57] | 12/699 | 58-74 | 1-4 | - | Tai Chi, routine exercise, Qigong, walking, Cardiovascular training, Brazilian samba, resistance training, balance, stretching, physiotherapy, Hatha Yoga, functional body weight training | 20-60 | 4-24 | 2-5 | Light and vigorous | Pharmacology, no intervention | SLP |
| Gilat, 2021 [64] | 50/2972 | 60-81 | 1.5-3.2 | 3.3-13.3 | Physiotherapy, such as treadmill,  water exercises, nordic walking, Tai Chi, balance, endurance, yoga, slackline, curve walking and fall prevention | 20-90 | 2-24 | 1-5 | - | No intervention, usual activities | MOV |
| Molina, 2021 [73] | 10/343 | 58.8-73.2 | 1-4 | 5.4-7.0 | Cycle ergometer/  strength training | 30-60 | 1-8/9-104 | 2-3 | 40-70%CF  30-90%RM | Education, usual activities, other activities | COG; BAL; MOT; MOB; STR |
| Ruiz-Gonzalez, 2021 [65] | 18/616 | 29-86 | 1-3 | - | Strength, cardiorespiratory, stretching, balance, functional, circuit | 30-60 | 4-12 | 3-5 | 60-85%CF | Usual activities | BDNF |
| Gamborg, 2022 [69] | 33/1266 | 59-76 | 1-4.35 | 0.1-11 | Strength training, aerobic training, other activities and modalities | 20-70 | 4-24 | 2-5 | 30-90%RM  50-85%CF | No intervention, usual activities | STR; BAL; MOT; CARD; SLP; ANX/DEP; MOB; MOV |
| SENSORY-MOTOR ACTIVITIES | | | | | | | | | | | |
| Lee, 2008 [94] | 35/338 | - | 1-3 | - | Tai Chi | 45-90 | 8-16 | 2-3 | - | Qigong, dance,  walking and elastic  band exercise | QOL; ANX/DEP; BAL; MOV; MOT; FALL; CARD; ADL |
| Toh, 2013 [95] | 8/1210 | 55-78.5 | 1-4 | 2 | Tai Chi | 60-90 | 4-24 | 1-5 | - | Tango, Foxtrot, RT, Qigong, no intervention | QOL; BAL; MOV; MOT; MOB; WLK; STR |
| Ni, 2014 [74] | 9/409 | 60.8-72 | 1-3 | 2.3-8.7 | Tai Chi | 30-60 | 4-24 | 1-5 | - | Qigong, dance, strength, walking, no intervention | QOL; BAL; MOV; FALL; MOB |
| Yang, 2014 [86] | 8/470 | 63-69 | 1-4 | - | Tai Chi | 40-60 | 4-24 | 2-4 | - | No intervention, walking, stretching/resistance training, Qigong, and exercise | BAL; MOV; MOT; MOB |
| Harris, 2015 [87] | 11/225 | 69.8* | 1-3 | 6.9 | Exergames | 30-60 | - | 1-3 | - | Other exercises, no intervention | BAL; MOT |
| Yang, 2015 [88] | 15/799 | 64.5-75.9 | 1-4 | - | Tai Chi, Qigong | 30-65 | 4-50 | 1-5 | - | Medication, aerobic, dance, walking, stretching, strength training | QOL; BAL; MOV; MOT; ADL |
| Zhou, 2015 [96] | 9/589 | 63(66*)69 | 1-4 | - | Tai Chi: Yang style | 30-60 | 4-24 | - | - | Walking, dance exercise, Qigong, strength training and muscular, endurance | QOL; COG; BAL; MOV; MOT |
| Dockx, 2016 [75] | 7/263 | 61.1-75.4 | 1-4 | - | Virtual reality | 30-60 | 4-12 | 2-5 | - | Physiotherapy, no intervention | QOL; COG; BAL; MOV; MOT; WLK; ADL |
| Cwiekala-Lewis, 2017 [97] | 11/548 | 63-68 | 1-4 | 3-9 | Tai Chi | - | 1-24 | 2-5 | - | Qigong, RT, aerobic, strength, stretching, dance | QOL; BRE; BAL; MOV; MOT; FALL; MOB; STR; CARD; ADL; FLX |
| Kwok, 2016 [21] | 10/406 | 60.8-71.2 | 1-4 | 5.2-9.2 | Tai Chi, Qigong, Yoga, Tango, modern dance, Waltz/Foxtrot | 60 | 8-16  1 study  =52 | 1-3 | - | Education or no intervention | COG; ANX/DEP; HUM; BRE; BAL; MOV; MOT; MOB; WLK; STR; CARD; ADL; FLX |
| Song, 2017 [76] | 15/755 | 54(67.5*)72 | 1-4 | - | Tai Chi/Qigong | 45-90 | 5-24 | 1-3 | - | Aerobic exercise, resistance training or stretching, walking, dancing, multimodal | QOL; COG; ANX/DEP; BAL; MOT; FALL; STR |
| McDonell, 2018 [77] | 4/84 | 63.4-67.3 | 1-3 | 4.2 | LSVT-BIG | 60 | 4 | 4 | - | General exercise, treadmill walking, Nordic walking, domestic exercise | MOV; MOT; WLK |
| Stickdorn, 2018 [98] | 3/1210 | 62.8-67.1 | 1.8-2.8 | - | Lee Silverman  Voice Treatment BIG (LSVT-BIG) | 60 | 4 | 4 | 75%CFmax. PSE-BORG (5/10) | Nordic walking, group therapy, stretching, running machine | MOT |
| Winser, 2018 [89] | 10/720 | 60-72 | - | - | Tai Chi | 30-60 | 4-24 | 1-5 | - | Multimodal exercise, walking | BAL; MOT; FALL |
| Garcia-Agundez, 2019 [99] | 9/383 | 61.7-71 | 1-3 | 4-9 | Exergames (Wii, Kinetic, WiiMove) | 15-60 | 4-12 | 2-5 | - | Usual home care, clinical supervision | QOL; COG; BAL; MOT; WLK; ADL |
| Liu, 2019 [78] | 5/355 | 40-85 | 1-4 | - | Tai Chi | 30-60 | 4-24 | 2-3 | - | RT, walking, stretching, no intervention | BAL; FALL; MOB |
| Santos, 2019 [90] | 5/156 | 60-70 | 1.2-2.5 | 6.4-7.9 | Nintendo Wii | 30-75 | 4-12 | 2-5 | - | Education for the prevention of falls, conventional exercises, functional electrical stimulation | QOL; BAL |
| Suárez-Iglesias, 2019 [91] | 8/149 | 57-73.5 | 1-3 | - | Pilates | 60 | 6-12 | 2-3 | PSE-BORG | Walking, calisthenics, aerobics, flexibility, conventional | QOL; BAL; MOB; STR; CARD; FLX |
| Alexandre de Assis, 2020 [79] | 6/153 | - | - | - | Limited progressive treadmill training, PNF, running machine, RGT | - | 3-8 | 3 | Low | Rest, no intervention | MOV; WLK |
| Chen, 2020 [80] | 7/325 | 57.1-67.5 | 1-4 | - | Qigong | 30-120 | 8-48 | 2-5  1 study =7 | - | Aerobic (cycle), walking, no intervention | BAL; MOT; WLK |
| Jin, 2019 [81] | 22/1199 | 40-86 | 1-4 | - | Tai Chi, Yoga, Qigong | 30-60 | 4-24 | 2-5 | - | Exercise, usual care, stretching, resistance, no exercise | QOL; ANX/DEP; BAL; MOT |
| Campo-Prieto, 2021 [102] | 7/156 | 57-78 | - | - | Virtual reality | 5-30 | 3-4 | 3 | - | - | MOT |
| Cugusi, 2021 [83] | 6/322 | 61-69.4 | - | - | Exergames | 30-60 | 6-12 | 2-5 | - | Usual activities | QOL |
| Elena, 2021 [84] | 14/548 | 63-75.2 | 1-4 | 4.5-10 | Exergames | 45-60 | 4-12 | 2-5 | 0 | Conventional physiotherapy | QOL; ADL; MOB; MOT; BAL; MOV |
| Garcia-Lopez, 2021 [93] | 10/537 | 64.3-75.4 | - | - | Virtual reality | 30-75 | 5-12 | 2-5 | - | Physiotherapy, treadmill, exercise | EQ; FALL |
| Kamieniarz, 2021 [100] | 26/835 | 60.8-78.5 | 1-4 | 2.9-9.1 | Tai Chi, Qigong | 30-90 | 5-24 | 1-5 | - | Tango, RT, elongation, Qigong, Tai Chi, treadmill, aerobics, dance | QOL; COG; ANX/DEP; SLP; BAL; MOV; FALL; MOB; WLK; ADL |
| Mailankody, 2021[101] | 7/278 | - | 1-2 | - | Yoga | - | 8-12 | 1-2 | - | Education, usual activities, strength training | BAL; MOT; MOV; ANX/DEP; QOL; COG; SLP |
| Suarez-Iglesias, 2022 [92] | 5/120 | 63.5-75 | 1-3 | - | Yoga | 45-60 | 8-12 | 2 | - | Usual activities, no intervention | MOV; MOB; FALL; STR; CARD; ANX/DEP; ADL; COG; SLP; WLB |
| Yu, 2021 [82] | 17/724 | 53-74 | - | - | Tai Chi | 40-60 | 8 | 2-5 | Low-Moderate | Usual activities, Qigong, resistance and strength training, flexibility and other routines | QOL; BAL; MOV; MOT |
| Lei, 2019 [85] | 12/539 | 50-85 | 1-4 | - | Virtual reality | 30-60 | 5-12 | 2-5 | - | Conventional exercises | BAL; MOB; QOL |
| Sevcenko, 2022 [103] | 8/337 | 61/74 | 2-3 | - | Virtual reality | - | 4-12 | 2-5 | - | Conventional exercises | MOB; MOV; BAL; MOT; ADL; COG; QOL |
| OTHER PROTOCOLS | | | | | | | | | | | |
| Lim, 2005 [138] | 24/626 | 25-88 | 1-4 | - | Walking, strength, stretching, balance: (1) auditory cue, (2) visual indication, (3) tactile suggestion, and (4) a combination of the types mentioned above | 30-60 | 3-6 | 2-5 | - | NR | BAL; MOV; WLK |
| Crizzle, 2006 [139] | 7/548 | 55-84 | 2-3 | - | Balance, resistance training (various types) | 45-60 | 4-14 | 2-3 | - | Physiotherapy, balance | QOL; BAL; MOV; MOB; STR; ADL; FLX |
| Kwakkle, 2007 [140] | 23/1063 | 56.8-75.7 | 1-4 | 2-12 | Training program involving daily activities, motor, balance, gait exercises, BWSTT, resistance training, Qigong, stretching, strengthening exercises, functional training | 20-120 | 3-23  1 study =52 | 1-3 (6-7) | - | NR, Qigong, exercise program: coordination, strength, mobility, relaxation and endurance | QOL; ANX/DEP; BAL; MOV; MOT; STR; CARD; ADL |
| Goodwin, 2008 [108] | 14/495 | 64-74.5 | 1-4 | - | Stretching, PRET, aerobic, force, BWSTT, balance Qigong | 20-90 | 4-12 | 1-3 | - | Sitting Karate, treadmill walking, balance training, physiotherapy, usual care | QOL; ANX/DEP; BAL; MOV; FALL; MOB; STR |
| Dibble, 2009 [141] | 16/596 | 62.5-76.2 | 1-4 | 4.8-11.2 | Whole body, vibration, balance, strength, treadmill, physical therapy, Tango, body weight support Mat, flexibility, Qigong | 15-90 | 3-12 | 3-7  1 study =10 | - | Board balance, calisthenic balance, treadmill, strength, musical, medical, no intervention | QOL; BAL; FALL |
| Allen, 2011 [109] | 16/2816 | 62.9-75.8 | - | - | Exercise and motor training = Highly challenging, balance training, task training (suggested), physical training, task training (motion strategy), strength training, stretching/ROM exercise, floor walking/exercise, treadmill | - | 2-13 | - | - | ND | BAL; FALL |
| de Dreu, 2012 [142] | 6/168 | 62.5-72.6 | 2-3 | - | Music-based movement therapy (MbM): walking, Tango, Waltz, Fox trot, Tai Chi | 30-60 | 3-13 | 2-3  1 study =7 | - | No intervention | QOL; BAL; MOV; MOT; WLK |
| Ayán Pérez, 2014 [121] | 12/199 | 56-89 | 1-3 | - | WBE intervention: aerobic capacity, balance and posture, control, mobility and strength | 30-60 | 4-20 | 1-3 | 11-14 PSE-BORG | Control group | QOL; COG; DIS; BAL; MOT; MOB; WLK; STR; CARD; ADL; FLX |
| Foster, 2014 [143] | 12/630 | 18-85 | 1-4 | - | Tango, strength, endurance, mobility, Foxtrot, Tai Chi, walking | - | 4-13 | 2-3 | - | No intervention | QOL; BAL; MOT; MOB; CARD |
| Mandelbaum, 2014 [144] | 10/295 | - | - | - | Dance: Argentinian Tango, modern Dande, improvisation, American ballroom | 60 | 2-52 | 1-2 | - | No intervention | QOL; ANX/DEP; BAL; MOV; MOT; FALL |
| Murray, 2014 [145] | 7/187 | 60-70 | - | - | Tango, aerobic, cycling, multimodal, Wii Fit, LIE-stretching, strength, Nordic walking | 60-90 | 4-24 | 1-3 | - | Education, usual care, no intervention | COG; HUM; BAL |
| Sharp, 2014 [122] | 5/199 | 63.3-71 | 2.1-2.6 | 4.9-8.9 | Dance: Tango, Foxtrot, Irish | 60-90 | 13-52 | 2 | - | Exercise, no intervention | QOL; BAL; MOV; MOT |
| Alves da Rocha, 2015 [146] | 32/1210 | 60.2-72.8 | - | 2.4-11.2 | Dance, hydrotherapy, Tai Chi, virtual reality, mental practice, aquatic exercise, aerobic exercise, robotic gait training, boxing, full body vibration, Nordic gait training | 20-90 | - | 1-5 | - | Conventional physiotherapy, treadmill, no intervention | QOL; DIS; BAL; MOV; FALL; MOB |
| Lötzke, 2015 [123] | 13/252 | 63-86 | 1-4 | - | Tango | 60-90 | 2-13  1 study  =104 | 2-5 | - | NR | QOL; COG; ANX/DEP; FAT; BAL; MOV; MOT |
| Shanahan, 2015 [147] | 9/410 | 61.6-74.4 | 1-2.5 | - | Dance: Tango, Irish dance | 60-90 | 10-52 | 1-2 | - | Educational group, physiotherapy, Tai Chi, exercise | QOL; BAL; MOT; MOB; CARD |
| Shen, 2016 [110] | 24/1881 | 61.8-73.2 | 1-4 | - | Balance training, gait, strength, LSVT-BIG, Nordic walking, another exercise | - | 2-48 | 1-4 | - | No intervention | BAL; MOV; FALL |
| Wang, 2016 [111] | 9/181 | 48-90 | 1-3 | - | Cognitive motor exercise | 60 | 4-13 | 2-3 | - | Balance training, regular activities, no intervention | BAL; MOV; MOB |
| Aguiar, 2016 [148] | 19/532 | 61.6-74.4 | 1-4 | 3.3-11 | Tango, Waltz, Foxtrot, contemporary dance, Irish dance, modern dance | 60-90 | 8-52  1 study =104 1-5 | 1-5 | - | Control, exercise, Tai Chi, education, ballet | QOL; MOV; MOB; WLK; CARD |
| Cassamatis, 2016 [124] | 6/240 | 64-76 | 1-4 | 8.1-9.3 | Cardiovascular warm-up, functional strengthening exercises, functional training, treadmill training through external auditory signaling, balance training, relaxation exercises, occupational therapy (OT), walking, aerobic training | 30-90 | 2-6 | 1-5 | - | Drug therapy, control of relaxation and breathing, musculoskeletal exercises, muscle strengthening exercises, muscle length and range of motion (exercise), aerobic training, education on the benefits of physical exercise and fall prevention | ADL |
| Cusso, 2016 [125] | 20/1009 | 40-89 | - | - | Aerobic training, Qigong, progressive resistance exercise, Tango, home exercise program, physiotherapy, treadmill speed training, Ronnie Gardiner Rhythm, balance training stretching and resistance training, Tai Chi, Nordic walking, walking, supported by body weight | 20-90 | 4-481  1 study =104  1 study =156 | 2-4 | - | Medication or not | COG; ANX/DEP; HUM; FAT; DIS; SLP; APA |
| Klamroth, 2016 [112] | 22/1072 | 58* | 1-4 | - | Multicomponent exercise program (at home), balance exercise, dancing, aerobic exercise, Nordic walking, cycling exercise, walking, Tai Chi, treadmill training, gait training | 40-60 | 4-104 | 2-3 | - | Usual care, Sham exercise (stretching), no exercise | BAL |
| McNeely, 2015 [149] | 7/210 | - | - | - | Tango, Waltz, Foxtrot, contemporary dance, Irish dance, modern dance | 60-90 | 10-104 | 1-2 | - | Physiotherapy, exercise controls, non-intervention, controls for education only | BAL; MOV; MOT; MOB; WLK; STR; CARD; FLX |
| Yitayeh, 2016 [126] | 8/483 | 63.3-75.7 | 2-4 | - | Physiotherapy stretching, aerobic training, muscle relaxation and activation, strength exercises and treadmill walking | 40-60 | 4-24 | 2-5 | - | Control group without intervention, nursing visit, mobilization and stretching, encouragement of physical activity, medication and activities at home | BAL; FALL |
| Delabary, 2018 [113] | 5/159 | 61.6-72.6 | 1-4 | - | Tango, Irish dance | 60-90 | 12-48 | 1-2 | - | - | QOL; MOV; MOT; MOB; WLK |
| Mazzarin, 2017 [127] | 9/485 | 55-72.6 | 1-4 | - | Tai Chi, Qigong, Irish dance, Tango | 60-90 | 24-54 | 2-3 | - | Physiotherapy, Qigong, resistance training, stretching, breathing, resistance | BAL; MOV; FALL; MOB |
| Wu, 2017 [150] | 9/342 | 30(65*)90 | 1-3 | - | Physical activity: aerobic training, VR Dance, Qigong, aerobic exercise (coordination, muscular endurance, balance, stretching, range of motion, mobility exercises, relaxation exercises, balance, coordination training, gait exercises and breathing exercises, LSVT BIG, Tai Chi, intensified training exercise for PD (Exceeded) | 20-60 | 4-24 | 2-5 | - | Maintenance of the same daily routine, no intervention | QOL; ANX/DEP; MOT |
| Connors, 2018 [128] | 21/151 | 70.6* | - | - | Functional exercise, cycling, LSVT, occupational therapy | - | 8-12 | 1.5 | - | - | QOL; COG; BAL; MOV; MOB |
| Costa, 2018 [151] | 3/102 | - | - | - | March on water | 45 | 3-16 | 2-5 | - | Conventional exercise | MOV; MOB |
| De Freitas, 2020 [152] | 7/259 | 61-72.9 | 2-3 | - | Dual Task Training | 25-60 | 4-10  1 study =30 | 1-3 | - | Simple task, no intervention | BAL; MOV |
| Caroll, 2020 [129] | 14/472 | 60.5-68.4 | 1.5-3 | - | Full body stretching, cardiovascular or relaxation activities, Tai Chi, aquatic task exercises, gait training exercises, aquatic therapy | 30-60 | 3-11 | 2-5  1 study  =7 | PSE-BORG | Physiotherapy, usual care | QOL; BAL; MOV; MOT; MOB |
| Chiong, 2019 [153] | 10/534 | 62.4-74.2 | 1-4 | - | Aquatherapy, resistance exercise | 30-120 | 5-24 | 2-5  1 study =1 |  | Life skills program + similar weekly home program, usual care, no exercise, land-based exercise, physiotherapy | QOL; COG; BAL; MOV; FALL; MOB |
| Cugusi, 2019 [105] | 7/187 | 61-71 | 1-3 | 9.2 | Aquatic exercise | 45-60 | 4-10 | 2-5 | - | Land exercise, control of usual medical therapy and normal daily activities | QOL; BAL; MOV; FALL |
| Flynn, 2019 [114] | 16/1700 | 60-74 | 1-4 | 5-9 | Home-Based: balance and strength, walking, treadmill walk | 15-60 | 3-26 | 2-7 | - | Usual care, placebo, or center-based exercise, no intervention | QOL; BAL; MOV |
| Kalyani, 2019 [115] | 27/586 | 68.1* | 1-3 | - | Irish dance, Tango | 60-90 | 2-104 | 1-5 | Energy expenditure, CF | Traditional exercise classes, home exercises or education sessions | COG; HUM; BAL; MOV; WLK |
| Morris, 2019 [130] | 2/37 | 60-68 | - | 2.3-4.2 | Boxe, exercise (strength, resistance, balance) | 45-90 | 12 | 2-3 | - | Exercise, no intervention | QOL; BAL; MOV; MOT; FALL; MOB |
| Perry, 2019 [104] | 10/1115 | - | 1-4 | - | Functional task training | 20-120 | 3-18 | 1-3(7) | - | Usual care, waiting, neuromotor, medication, physical incentive | QOL; BAL; MOV; MOT; FALL; ADL |
| Pinto, 2019 [106] | 19/484 | 54-78 | 1-4 | 3-10 | Hydrotherapy | 40-60 | 3-20 | 1-5 | - | Usual care, medication and routine activities, earth-based therapy, water walking therapy, aquatic therapy obstacle | QOL; BAL; MOT; MOB |
| Pritchard, 2019 [131] | 10/332 | 62.4-78.4 | 1-3 | - | Aquatic therapy | 40-60 | 4-12 | 2-5 | - | UW; LBW; MIRT, usual care | QOL; BAL; MOV |
| Pupíková, 2020 [154] | 10/415 | 64-73 | 1.6-2.8 | 4.2-11 | Aerobic stationary cycling, goal-based exercise modalities, aerobic exercise – treadmill, stretching and balance training, proprioceptive and strengthening + balance and gait training during cognitive tasks, dance, rehabilitation exercise, multimodal exercise (muscular resistance, motor coordination, balance), Tai Chi, resistance training (RT), resistance training with instability (RTI), “HiBalance” program (dual-task training), virtual reality balance training, conventional balance training | - | 4-24 | 1-3 | - | NR | COG |
| Zhang, 2019 [116] | 7/185 | 63.2-68.4 | 2.1 | 5.5-7 | Tango, aerobic, Jazz, Ballet, VRD, dance | 30-90 | 6-12 | 1-5 | - | No dance, usual home-activities, no intervention | COG; ANX/DEP; APA |
| Barnish, 2020 [134] | 38/1064 | 61-74 | 1-4 | - | Specific dance, Tango-based dance, modern dance, Waltz/Foxtrot, Ballet | 60-90 | 3-32 | 1-5 | - | Education, exercise music enjoyment,  painting, dancing or Tai Chi, physiotherapy, support group, usual care, traditional rehabilitation, running machine, stretching | QOL; COG; MOT |
| Consentino, 2020 [135] | 19/913 | *69.6 | 1-4 | 9.4 | Physiotherapy | 23-60 | 4-24 | 2-3 | - | No intervention, usual activities | MOV; QOL; MOT |
| Neto, 2020 [133] | 15/462 | 61.8-71.4 | - | - | Water-based exercise | 45-60 | 4-10 | 1-5 | - | Ground exercise | QOL; BAL; MOT; FALL; MOB; ADL |
| Hidalgo-Agudo, 2020 [134] | 5/1210 | - | 1-3 | - | Aquatic physiotherapy, dance-based therapy | 60-90 | 8-24 | 1-5 | - | NR | QOL; BAL; MOT; FALL |
| Li, 2020 [26] | 11/322 | 59-73 | 1-4 | - | Double task | 20-60 | 3-48 | 1-4 | - | No intervention, usual activities, education | MOV; MOT; BAL |
| Miller, 2020 [117] | 4/138 | 61.6-81.4 | 1-4 | - | Hydrotherapy, equilibrium, dance, treadmill, force | 20-30 | 2-52 | 2-5 | - | Usual activities | MOV |
| Radder, 2020 [155] | 191/7998 | 30-90 | 1-4 | 0.3-18.7 | Conventional physical therapy, resistance training, treadmill training, strategy training, dance, martial arts, aerobic exercise, hydrotherapy, balance and gait training, dual task, exergaming and Nordic walking | *67 | 4-104 | 1-7 | - | No intervention, usual activities | MOT; BAL; MOV; QOL |
| Abou, 2021 [107] | 25/1705 | 37-78 | 1-4 | 1.4-10 | Conventional physical therapy, resistance training, treadmill training, strategy training, dance, aerobic exercise, balance and gait training, Tai Chi | 15-135 | 3-55 | 1-7 | - | No intervention, usual activities, education | FALL |
| Foster, 2021 [156] | 13/1512 | 40-75 | 1-4 | - | Resistance training, treadmill training, dance, aerobic exercise, hydrotherapy, balance and gait training, exergaming and Nordic walking | 30-70 | 4-24 | 1-3 | - | No intervention, usual activities, education | ADL |
| Ismail, 2021 [118] | 18/723 | 50-90 | 1-4 | - | Dances | 60-90 | 8-48 | 1-3 | - | Physiotherapy, usual activities, education, exercises | BAL; MOV; MOT; MOB; ANX/DEP; WAL; HUM; FAT; APA; QOL; COG; FALL |
| Oh, 2021 [136] | 3/88 | 62.4-77 | - | - | Aquatic activities | 45-60 | 5-8 | 1-5 | - | Usual activities on the ground | MOB; MOT; BAL; QOL |
| Okada, 2021 [119] | 10/448 | - | 1-3 | - | Aerobic, strength, multimodal, physiotherapy | 30-90 | 4-104 | 2-7 | - | Usual activities, exercise, no intervention | MOT; QOL; ADL; WAL; MOV; CARD; COG; SLP; ANX/DEP |
| Zhou, 2021 [120] | 17/598 | 61.6-74 | 1-3 | 4.4-9 | Music-based movement | 30-120 | 3-48 | 1-7 | - | Usual activities, education, no intervention | MOV; MOT; BAL; QOL; ANX/DEP |
| Hasan, 2022 [137] | 14/372 | 61.6-75.5 | 1-4 | 4.4-9.2 | Dances | 30-90 | 6-48 | 1-2 | - | Physiotherapy, exercise, no intervention | BAL; MOB; MOV; MOT; ADL; WAL; APA; COG; ANX/DEP; FAT; QOL |

* Mean age

NR – not reported; CF – cardiac frequency; CFmax – maximum cardiac frequency; CFreserve – reserve cardiac frequency; MR – maximum repetition; RPE-BORG – Rate of Perceived Exertion; RPM – rotations per minute; km/h – kilometers per hour; ME – maximum expiration; MI – maximum inspiration.

QOF – Quality of life; FAT – Fatigue; EQ – Equilibrium; MOV – Movement/walking; MOTF – Motor function; FALL – Falls; CARD – cardiorespiratory; MOB – mobility; WLK – walking; COG – cognitive function; ANX/DEP – anxiety/depression; BDNF – brain-derived neurotrophic factor; ADL – activities of daily living; DIS – disease severity; APA – apathy; STR – strength; HUM – humor; SLP – sleep; WLB – well-being; FLX – flexibility
