# Supplementary material for: Physical exercise and its effects on people with Parkinson’s disease: Umbrella review
Source: PLoS One. 2023 Nov 2;18(11):e0293826. doi: 10.1371/journal.pone.0293826 (PMC10621990; doi:10.1371/journal.pone.0293826)
Supplement: S3 Table — (DOCX) [file pone.0293826.s003.docx]

**S3 Table. AMSTAR 2 assessment of the included systematic reviews**

|  | **QUESTIONS** | | | | | | | | | | | | | | | | **DOMAINS** | | | | | | |  |
| --- | --- | --- | --- | --- | --- | --- | --- | --- | --- | --- | --- | --- | --- | --- | --- | --- | --- | --- | --- | --- | --- | --- | --- | --- |
|  | **Q1** | **Q2** | **Q3** | **Q4** | **Q5** | **Q6** | **Q7** | **Q8** | **Q9** | **Q10** | **Q11** | **Q12** | **Q13** | **Q14** | **Q15** | **Q16** | **D1** | **D2** | **D3** | **D4** | **D5** | **D6** | **D7** | **AMSTAR QUALITY RATING*** |
| Lim, 2005 | Y | N | Y | PY | Y | Y | N | Y | Y | N | N/A | N/A | Y | N | N | N | N | Y | N | Y | N | N | N | Critically low |
| Crizzle, 2006 | Y | N | Y | PY | N | N | N | PY | N | N | N/A | N/A | N | N | N | N | N | Y | N | N | N | N | N | Critically low |
| Kwakkel, 2007 | Y | N | Y | PY | Y | Y | N | Y | Y | N | N/A | N/A | N | N | N | Y | N | Y | N | Y | N | Y | N | Critically low |
| Goodwin, 2008 | Y | N | Y | PY | N | Y | PY | Y | Y | N | Y | Y | Y | Y | N | Y | N | Y | Y | Y | Y | Y | N | Moderate |
| Lee, 2008 | Y | N | Y | Y | Y | Y | PY | Y | Y | N | N/A | N/A | Y | N | N | N | N | Y | Y | Y | N | N | N | Critically low |
| Dibble, 2009 | Y | N | N | PY | Y | Y | PY | Y | N | N | N/A | N/A | N | N | N | N | N | Y | N | N | N | N | N | Critically low |
| Herman, 2009 | N | N | N | N | N | N | N | Y | N | N | N/A | N/A | N/A | N/A | N/A | N | N | N | N | N | N | N | N | Critically low |
| Allen, 2011 | Y | N | Y | N | Y | Y | PY | Y | Y | N | Y | Y | Y | Y | Y | Y | N | Y | Y | Y | Y | Y | Y | Moderate |
| Jambeau, 2011 | Y | N | Y | PY | N | N | PY | Y | N | N | N/A | N/A | N | N | N | N | N | Y | Y | N | N | N | N | Critically low |
| de Dreu, 2012 | Y | N | Y | PY | Y | Y | PY | Y | Y | N | Y | Y | Y | Y | Y | N | N | Y | N | Y | Y | Y | N | Critically low |
| Brienesse, 2013 | Y | N | Y | PY | Y | Y | PY | PY | PY | N | N | N | N | N | N | N | N | Y | N | Y | Y | Y | N | Low |
| Lima, 2013 | Y | Y | Y | Y | Y | Y | PY | Y | Y | N | Y | Y | Y | Y | N | N | Y | Y | Y | Y | Y | Y | N | Moderate |
| Toh, 2013 | N | N | Y | PY | N | N | N | Y | PY | N | N/A | N/A | Y | N/A | N/A | Y | N | N | N | Y | N | N | N | Critically low |
| Ayán, 2014 | Y | N | Y | PY | Y | Y | N | Y | Y | N | N/A | N/A | Y | N | N | N | N | Y | Y | Y | N | Y | N | Low |
| Foster, 2014 | Y | N | N | N | N | N | N | Y | N | N | N/A | N/A | N | N | N | N | N | Y | N | N | N | N | N | Critically low |
| Mandelbaum, 2014 | Y | N | N | PY | N | N | PY | PY | N | N | N/A | N/A | N | N | N | N | N | Y | Y | N | N | N | N | Critically low |
| Murray, 2014 | Y | PY | Y | PY | Y | Y | PY | PY | PY | N | N/A | N/A | Y | N | N | Y | N | Y | Y | Y | N | N | N | Critically low |
| Ni, 2014 | Y | Y | Y | Y | Y | Y | Y | Y | Y | N | Y | Y | Y | Y | Y | Y | Y | Y | Y | Y | Y | Y | Y | High |
| Sharp, 2014 | Y | N | Y | Y | Y | Y | PY | Y | Y | N | Y | Y | Y | Y | N | N | N | Y | N | Y | Y | Y | N | Low |
| Shu, 2014 | Y | N | Y | Y | Y | Y | PY | Y | Y | N | Y | Y | Y | Y | N | Y | N | Y | Y | Y | Y | Y | N | Low |
| Tambosco, 2014 | Y | N | N | N | N | N | N | Y | N | N | N/A | N/A | N/A | N/A | N/A | Y | N | N | N | N | N | N | N | Critically low |
| Yang, 2014 | Y | PY | Y | Y | Y | Y | PY | Y | PY | N | Y | Y | Y | Y | N | Y | N | Y | Y | Y | Y | Y | N | Low |
| Alves da Rocha, 2015 | Y | N | Y | PY | Y | Y | PY | N | Y | N | Y | Y | Y | N | N | Y | N | Y | N | Y | N | Y | N | Critically low |
| Chung, 2015 | Y | N | Y | PY | Y | Y | PY | Y | Y | N | Y | Y | Y | Y | N | Y | N | Y | Y | Y | Y | Y | N | Low |
| Cruickshank, 2015 | Y | N | Y | PY | Y | Y | PY | Y | Y | N | Y | Y | Y | Y | Y | Y | N | Y | Y | Y | Y | Y | Y | Moderate |
| Harris, 2015 | Y | PY | Y | PY | Y | Y | PY | PY | Y | N | Y | Y | Y | Y | N | Y | N | Y | Y | Y | Y | Y | N | Low |
| Lamotte, 2015 | Y | N | N | PY | Y | Y | N | PY | N | N | Y | N | N | N | N | Y | N | Y | N | N | Y | N | N | Critically low |
| Lötzke, 2015 | Y | PY | Y | PY | Y | Y | PY | PY | Y | N | Y | Y | Y | Y | N | Y | N | Y | Y | Y | Y | Y | N | Low |
| Mehrholz, 2015 | Y | Y | Y | Y | Y | Y | Y | Y | Y | N | Y | Y | Y | Y | Y | Y | Y | Y | Y | Y | Y | Y | Y | High |
| Roeder, 2015 | Y | PY | Y | Y | Y | Y | Y | Y | Y | N | Y | Y | Y | Y | N | Y | N | Y | Y | Y | Y | Y | N | Low |
| Shanahan, 2015 | Y | N | Y | PY | Y | N | PY | Y | Y | N | N | N | Y | N | N | N | N | Y | N | Y | N | N | N | Critically low |
| Shen, 2015 | Y | N | Y | PY | Y | Y | PY | Y | Y | N | Y | Y | Y | Y | N | Y | N | Y | Y | Y | Y | Y | Y | Moderate |
| Tillman, 2015 | Y | PY | Y | PY | N | Y | PY | Y | Y | N | Y | Y | N | Y | N | Y | N | Y | N | Y | Y | N | N | Low |
| Uhrbrand, 2015 | Y | PY | Y | PY | N | N | PY | Y | Y | N | Y | Y | Y | Y | N | N | N | Y | Y | Y | Y | Y | N | Moderate |
| Wang, 2015 | Y | Y | Y | PY | Y | Y | PY | Y | Y | N | Y | Y | Y | Y | N | Y | Y | Y | Y | Y | Y | Y | N | Moderate |
| Yang, 2015 | Y | PY | Y | PY | Y | Y | PY | Y | Y | N | Y | Y | Y | Y | N | Y | N | Y | Y | Y | Y | Y | N | Low |
| Zhou, 2015 | Y | PY | Y | PY | Y | Y | PY | PY | N | N | Y | N | N | N | N | Y | N | N | N | N | Y | N | N | Critically low |
| Aguiar, 2016 | Y | N | Y | PY | Y | N | PY | Y | Y | N | N/A | N/A | N | N | N | N | N | Y | N | Y | N | N | N | Critically low |
| Cassimatis, 2016 | Y | N | Y | Y | Y | Y | PY | Y | Y | N | Y | Y | Y | Y | N | N | N | Y | Y | Y | Y | Y | N | Low |
| Cusso, 2016 | Y | Y | Y | PY | Y | Y | PY | Y | Y | N | N/A | N/A | Y | N | N | Y | Y | Y | Y | Y | N | Y | N | Low |
| Cwiekała-Lewis, 2016 | Y | N | Y | PY | N | Y | PY | Y | Y | N | N/A | N/A | N | N | N | Y | N | Y | Y | Y | N | N | N | Critically low |
| da Silva, 2016 | Y | Y | Y | PY | Y | Y | PY | Y | Y | N | N/A | N/A | Y | Y | N | N | Y | Y | Y | Y | N | Y | N | Low |
| Dockx, 2016 | Y | Y | Y | Y | Y | Y | Y | Y | Y | Y | Y | Y | Y | Y | N | Y | Y | Y | Y | Y | Y | Y | N | Moderate |
| Klamroth, 2016 | Y | N | Y | PY | Y | Y | Y | Y | Y | N | Y | Y | Y | Y | Y | Y | N | Y | Y | Y | Y | Y | Y | Moderate |
| Kwok, 2016 | Y | PY | Y | PY | Y | Y | PY | Y | Y | N | Y | Y | Y | Y | N | Y | N | Y | Y | Y | Y | Y | N | Low |
| McNeely, 2016 | Y | N | Y | PY | N | N | Y | PY | N | N | N | N | N | N | N | Y | N | Y | Y | N | N | N | N | Critically low |
| Reynolds, 2016 | Y | N | Y | PY | N | N | N | N | N | N | N/A | N/A | N | N | N | N | N | Y | N | N | N | N | N | Critically low |
| Saltychev, 2016 | Y | Y | Y | Y | Y | N | PY | Y | Y | N | Y | Y | Y | Y | Y | Y | Y | Y | Y | Y | Y | Y | Y | High |
| Silva, 2016 | Y | Y | Y | PY | Y | Y | PY | Y | Y | N | N/A | N/A | Y | N/A | N | Y | Y | Y | Y | Y | N | Y | N | Low |
| Yitayeh, 2016 | Y | PY | Y | PY | Y | Y | PY | Y | Y | N | Y | Y | Y | Y | N | Y | N | Y | Y | Y | Y | Y | N | Low |
| Bombieri, 2017 | Y | N | Y | PY | Y | Y | N | PY | N | N | N | N | N | Y | N | N | N | Y | N | N | N | N | N | Critically low |
| dos Santos Delabary, 2017 | Y | Y | Y | PY | Y | Y | PY | PY | Y | N | Y | Y | Y | Y | N | Y | Y | Y | Y | Y | Y | Y | N | Moderate |
| Flach, 2017 | Y | N | Y | PY | Y | Y | PY | Y | N | N | N | N | N | Y | N | Y | N | Y | Y | N | Y | N | N | Critically low |
| Mackay, 2017 | Y | Y | Y | Y | Y | Y | Y | Y | Y | N | Y | Y | N | Y | N | Y | Y | Y | Y | Y | Y | Y | N | Moderate |
| Mazzarin, 2017 | Y | PY | Y | PY | Y | Y | PY | Y | Y | N | Y | Y | Y | Y | N | Y | N | Y | Y | Y | Y | Y | N | Low |
| Ramazzina, 2017 | Y | PY | Y | Y | Y | Y | PY | Y | Y | N | N/A | N/A | Y | Y | N | N | N | Y | Y | Y | N | Y | N | Low |
| Song, 2017 | Y | N | Y | PY | Y | Y | PY | Y | Y | N | Y | Y | Y | Y | Y | Y | N | Y | Y | Y | Y | Y | Y | Moderate |
| Wu, 2017 | Y | PY | Y | PY | N | Y | PY | PY | Y | N | N/A | N/A | Y | N/A | N/A | Y | N | Y | Y | Y | N | Y | N | Critically low |
| Alwardat, 2018 | Y | PY | Y | PY | Y | Y | N | PY | Y | N | Y | Y | Y | Y | N | N | N | Y | N | Y | Y | Y | N | Low |
| Connors, 2018 | Y | Y | Y | Y | Y | Y | PY | PY | PY | N | N/A | N/A | Y | N | N | Y | Y | Y | Y | Y | N | Y | N | Low |
| Costa, 2018 | Y | N | Y | PY | N | N | N | N | Y | N | N/A | N/A | Y | N | N | Y | N | Y | Y | Y | N | Y | N | Critically low |
| da Silva, 2018 | Y | Y | Y | PY | Y | Y | PY | Y | Y | N | N/A | N/A | Y | Y | N | Y | Y | Y | Y | Y | N | Y | N | Low |
| de Freitas, 2018 | Y | Y | Y | PY | Y | Y | Y | Y | PY | N | N/A | N/A | N | N | N | N | Y | Y | Y | N | N | Y | N | Critically low |
| Hirsch, 2018 | Y | PY | Y | PY | Y | Y | PY | Y | Y | N | Y | Y | Y | Y | N | Y | N | Y | Y | Y | Y | Y | N | Low |
| McDonnell, 2018 | Y | Y | Y | PY | Y | Y | PY | Y | Y | N | Y | Y | Y | Y | N | Y | Y | Y | Y | Y | Y | Y | N | Moderate |
| Ni, 2018 | Y | N | Y | PY | N | Y | PY | PY | Y | N | Y | Y | Y | Y | Y | N | N | Y | Y | Y | Y | Y | Y | Moderate |
| Stickdorn, 2018 | Y | N | Y | PY | Y | Y | N | PY | Y | N | N/A | N/A | N/A | N | N | N | N | Y | Y | Y | N | N | N | Critically low |
| Winser, 2018 | Y | PY | Y | Y | Y | Y | Y | Y | Y | N | Y | Y | Y | Y | N | Y | N | Y | Y | Y | Y | Y | N | Low |
| Carroll, 2019 | Y | Y | Y | Y | Y | Y | PY | Y | Y | N | N | Y | Y | N | N | N | Y | Y | N | Y | Y | Y | N | Low |
| Chiong, 2019 | Y | N | Y | PY | N | N | N | Y | N | N | N/A | N/A | N | N | N | N | N | Y | N | N | N | N | N | Critically low |
| Cugusi, 2019 | Y | Y | Y | PY | Y | Y | Y | Y | Y | N | Y | Y | Y | Y | Y | Y | Y | Y | Y | Y | Y | Y | Y | High |
| Flynn, 2019 | Y | Y | Y | PY | Y | Y | PY | Y | Y | N | Y | Y | Y | Y | N | Y | Y | Y | Y | Y | Y | Y | N | Moderate |
| Garcia-Agundez, 2019 | Y | N | Y | PY | N | N | PY | Y | N | N | N/A | N/A | N | N | N | Y | N | Y | Y | N | N | N | N | Critically low |
| Kalyani Haputhanthirige, 2019 | Y | Y | Y | PY | Y | Y | Y | Y | Y | N | Y | Y | Y | Y | N | Y | Y | Y | Y | Y | Y | Y | N | Moderate |
| Liu, 2019 | Y | Y | Y | Y | Y | Y | PY | Y | Y | N | Y | Y | Y | Y | N | Y | Y | Y | Y | Y | Y | Y | N | Moderate |
| Morris, 2019 | Y | Y | Y | Y | Y | Y | Y | Y | Y | N | N | N | Y | Y | N | Y | Y | Y | Y | Y | N | Y | N | Low |
| Perry, 2019 | Y | Y | Y | PY | Y | Y | PY | Y | Y | N | Y | Y | Y | Y | Y | N | Y | Y | Y | Y | Y | Y | Y | High |
| Pinto, 2019 | Y | Y | Y | Y | Y | Y | PY | Y | Y | N | Y | Y | Y | Y | Y | N | Y | Y | Y | Y | Y | Y | Y | High |
| Pritchard, 2019 | Y | PY | Y | Y | Y | Y | PY | Y | Y | N | N | Y | Y | Y | N | N | N | Y | Y | Y | N | Y | N | Low |
| Pupíková, 2019 | Y | N | Y | PY | N | N | PY | Y | N | N | N | N | N | Y | N | N | N | Y | Y | N | N | Y | N | Critically low |
| Robinson, 2019 | Y | Y | Y | Y | Y | Y | PY | Y | Y | N | Y | Y | Y | Y | Y | Y | Y | Y | Y | Y | Y | Y | Y | High |
| Rodriguez, 2019 | Y | PY | Y | PY | Y | Y | PY | Y | Y | N | N/A | N/A | N | Y | N | Y | N | Y | Y | Y | N | N | N | Critically low |
| Santos, 2019 | Y | PY | Y | PY | Y | Y | PY | PY | Y | N | Y | Y | Y | Y | N | N | N | Y | N | Y | Y | Y | N | Low |
| Seuthe, 2019 | Y | N | Y | PY | N | N | PY | PY | N | N | N/A | N/A | N | N | N | Y | N | Y | N | N | N | N | N | Critically low |
| Stuckenschneider, 2019 | Y | Y | Y | Y | N | Y | PY | Y | Y | N | Y | Y | Y | N | N | Y | Y | Y | Y | Y | Y | Y | N | Moderate |
| Suárez-Iglesias, 2019 | Y | Y | Y | PY | Y | N | PY | Y | Y | N | Y | Y | N | Y | N | Y | Y | Y | Y | Y | Y | N | N | Low |
| Zhang, 2019 | Y | PY | Y | PY | Y | Y | PY | Y | Y | N | Y | Y | Y | Y | Y | Y | N | Y | Y | Y | Y | Y | Y | Moderate |
| da Costa, 2020 | Y | N | Y | PY | N | N | PY | Y | Y | N | N/A | N/A | Y | N | N | N | N | Y | Y | Y | N | Y | N | Low |
| Aburub, 2020 | Y | N | Y | PY | N | N | PY | PY | Y | N | N/A | N/A | Y | N | N | Y | N | Y | N | Y | N | Y | N | Critically low |
| Alexandre de Assis, 2020 | Y | Y | Y | PY | Y | Y | PY | PY | Y | N | Y | Y | Y | Y | N | Y | Y | Y | Y | Y | Y | Y | N | Moderate |
| Barnish, 2020 | Y | PY | Y | PY | Y | Y | PY | Y | PY | N | Y | Y | Y | Y | N | Y | N | Y | Y | Y | Y | Y | N | Low |
| Chen, 2020a | Y | Y | Y | Y | Y | Y | PY | Y | Y | N | Y | Y | Y | Y | Y | Y | Y | Y | Y | Y | Y | Y | Y | High |
| Chen, 2020b | Y | PY | Y | PY | Y | Y | PY | Y | Y | N | Y | Y | Y | Y | Y | Y | N | Y | Y | Y | Y | Y | Y | Moderate |
| Choi, 2020 | Y | PY | Y | Y | Y | Y | Y | Y | Y | N | Y | Y | Y | Y | N | Y | N | Y | Y | Y | Y | Y | N | Moderate |
| Consentino, 2020 | Y | Y | Y | PY | Y | Y | PY | PY | Y | N | Y | Y | Y | Y | N | Y | Y | N | N | Y | Y | Y | N | Low |
| De Santis, 2020 | Y | Y | Y | PY | Y | Y | N | Y | Y | N | N/A | N/A | Y | N | N | Y | Y | Y | Y | Y | N | Y | N | Low |
| Gomes Neto, 2020 | Y | PY | Y | PY | Y | Y | PY | N | Y | N | Y | Y | Y | Y | N | Y | N | Y | Y | Y | Y | Y | N | Low |
| Hidalgo-Agudo, 2020 | Y | PY | Y | PY | Y | Y | PY | Y | Y | N | Y | Y | Y | Y | N | Y | N | Y | Y | Y | Y | Y | N | Low |
| Jin 2020 | Y | N | Y | PY | Y | Y | Y | Y | Y | N | Y | Y | Y | Y | Y | Y | N | Y | Y | Y | Y | Y | Y | Moderate |
| Johansson, 2020 | Y | Y | Y | PY | Y | Y | PY | Y | Y | N | Y | N | Y | Y | Y | Y | Y | Y | Y | Y | Y | N | Y | Moderate |
| Li, 2020a | Y | N | Y | Y | Y | Y | Y | Y | Y | Y | Y | Y | Y | Y | Y | Y | Y | N | Y | Y | Y | Y | Y | Moderate |
| Li, 2020b | Y | PY | Y | Y | Y | Y | PY | Y | Y | N | Y | Y | Y | Y | Y | Y | N | Y | Y | Y | Y | Y | Y | Moderate |
| Li, 2020 | Y | Y | Y | PY | Y | Y | PY | Y | Y | N | Y | Y | Y | Y | Y | Y | Y | N | Y | Y | Y | Y | Y | Moderate |
| McMahon, 2020 | Y | Y | Y | Y | Y | Y | Y | Y | Y | N | Y | Y | Y | Y | N | Y | Y | Y | Y | Y | Y | Y | N | Moderate |
| Miller 2020 | Y | Y | Y | PY | Y | Y | PY | Y | Y | N | Y | Y | Y | Y | Y | N | Y | Y | N | Y | Y | Y | Y | Moderate |
| Miner, 2020 | Y | N | N | PY | N | N | PY | Y | N | N | N/A | N/A | N | N | N | N | N | Y | N | N | N | N | N | Critically low |
| Radder, 2020 | Y | PY | Y | PY | Y | Y | PY | PY | N | N | Y | N | N | Y | Y | Y | N | N | Y | N | Y | N | N | Critically low |
| Rodriguez, 2020 | Y | PY | Y | PY | Y | Y | PY | Y | Y | N | N/A | N/A | N | N/A | N | Y | N | Y | Y | Y | N | Y | N | Low |
| Smith, 2020 | N | Y | Y | PY | Y | Y | PY | Y | Y | N | N/A | N/A | Y | Y | N/A | Y | Y | Y | N | Y | N | Y | N | Critically low |
| van de Wetering, 2020 | Y | PY | Y | PY | Y | Y | PY | PY | Y | N | N/A | N/A | N | N/A | N/A | Y | N | Y | Y | Y | N | N | N | Low |
| Abou, 2021 | Y | Y | Y | PY | Y | Y | PY | Y | Y | N | Y | Y | Y | Y | Y | N | Y | Y | Y | Y | Y | Y | Y | High |
| BRAZ, 2021a | Y | PY | Y | PY | Y | Y | Y | Y | PY | N | N/A | N/A | Y | Y | N/A | N | N | Y | N | Y | N | N | N | Critically low |
| BRAZ, 2021b | Y | PY | Y | PY | Y | Y | PY | Y | PY | N | N/A | N/A | Y | Y | N/A | Y | N | Y | N | Y | N | Y | N | Critically low |
| Campo-Prieto, 2021 | Y | PY | Y | PY | Y | Y | PY | PY | N | N | N/A | N/A | N | N | N/A | N | N | Y | Y | N | N | N | N | Critically low |
| Cristini, 2021 | Y | Y | Y | PY | Y | Y | PY | Y | Y | N | Y | Y | Y | Y | Y | Y | Y | Y | Y | Y | Y | Y | Y | High |
| Cugusi, 2021 | Y | PY | Y | PY | N | Y | PY | PY | Y | N | Y | Y | Y | Y | Y | Y | N | Y | Y | Y | Y | Y | Y | Moderate |
| Elena, 2021 | Y | Y | Y | PY | Y | Y | PY | Y | Y | N | Y | Y | Y | Y | N | Y | Y | Y | Y | Y | Y | Y | N | Moderate |
| Foster, 2021 | Y | PY | Y | PY | Y | Y | PY | Y | Y | N | N/A | N/A | Y | N | N/A | Y | N | N | N | Y | N | Y | Y | Critically low |
| Garcia-Lopez, 2021 | Y | Y | Y | Y | Y | Y | PY | PY | Y | N | N/A | N/A | Y | N | N/A | Y | Y | Y | Y | Y | N | N | N | Low |
| Gilat, 2021 | Y | Y | Y | PY | Y | Y | PY | Y | Y | N | Y | Y | Y | Y | Y | Y | Y | Y | N | Y | Y | Y | Y | Moderate |
| Ismail, 2021 | Y | Y | Y | PY | Y | Y | PY | Y | Y | N | Y | Y | Y | Y | Y | Y | Y | Y | N | Y | Y | Y | Y | Moderate |
| Kamieniarz, 2021 | Y | N | Y | Y | Y | Y | PY | Y | Y | N | N/A | N/A | Y | N | N | Y | N | Y | Y | Y | N | Y | N | Critically low |
| Lorenzo Gracia, 2021 | Y | Y | Y | Y | Y | Y | PY | Y | Y | N | Y | Y | Y | Y | Y | Y | Y | Y | Y | Y | Y | Y | Y | High |
| Mailankody, 2021 | N | PY | Y | N | Y | Y | PY | PY | PY | N | N/A | N/A | N | Y | N/A | Y | N | N | N | Y | N | Y | N | Critically low |
| Molina, 2021 | Y | N | Y | PY | N | N | PY | Y | PY | N | N/A | N/A | N | N | N/A | Y | N | Y | Y | Y | N | Y | N | Critically low |
| OH, 2021 | Y | PY | Y | PY | Y | Y | PY | PY | Y | N | Y | Y | Y | Y | N | Y | N | N | Y | Y | Y | Y | N | Low |
| Okada, 2021 | Y | Y | Y | PY | Y | Y | PY | Y | Y | N | Y | Y | Y | Y | Y | Y | Y | N | Y | Y | Y | Y | Y | Moderate |
| Palheta, 2021 | Y | Y | Y | PY | Y | Y | PY | PY | N | N | N/A | N/A | N | N | N/A | N | Y | Y | N | N | N | N | N | Critically low |
| Ruiz-Gonzalez, 2021 | Y | Y | Y | Y | Y | Y | PY | Y | Y | N | Y | Y | Y | Y | Y | Y | Y | Y | N | Y | Y | Y | Y | Moderate |
| Suarez-Iglesias, 2021 | Y | PY | Y | PY | Y | Y | PY | PY | Y | N | Y | Y | Y | Y | N | Y | N | N | Y | Y | Y | Y | N | Low |
| Tiihonen, 2021 | Y | Y | Y | N | Y | Y | PY | Y | PY | N | Y | Y | Y | Y | Y | Y | Y | N | Y | Y | Y | Y | Y | Moderate |
| Wang, 2021 | Y | PY | Y | PY | Y | Y | PY | Y | Y | N | Y | Y | Y | Y | Y | Y | N | N | Y | Y | Y | Y | Y | Moderate |
| Yu, 2021 | Y | PY | Y | PY | N | Y | PY | Y | Y | N | Y | Y | Y | Y | Y | Y | N | Y | Y | Y | Y | Y | Y | Moderate |
| Zhou, 2021 | Y | PY | Y | PY | Y | Y | PY | Y | Y | N | Y | Y | Y | Y | Y | Y | N | N | Y | Y | Y | Y | Y | Moderate |
| De Almeida, 2022 | Y | PY | Y | PY | Y | Y | PY | Y | Y | N | Y | Y | Y | Y | Y | Y | N | Y | Y | Y | Y | Y | Y | Moderate |
| Gamborg, 2022 | Y | PY | Y | PY | Y | Y | Y | Y | PY | N | Y | Y | Y | Y | N | Y | N | Y | Y | Y | Y | Y | N | Low |
| Hasan, 2022 | Y | PY | Y | PY | Y | Y | PY | Y | Y | N | Y | Y | Y | Y | N | Y | N | N | Y | Y | Y | Y | N | Low |
| Salse-Batán, 2022 | Y | PY | Y | PY | N | Y | PY | Y | Y | N | Y | Y | Y | Y | N | Y | N | Y | N | Y | Y | Y | N | Low |
| Sevcenko, 2022 | Y | PY | Y | PY | N | N | PY | PY | PY | N | N/A | N/A | N | Y | N/A | Y | N | N | N | Y | N | Y | N | Critically low |

*** AMSTAR quality rating: High; Moderate; Low; Critically low**

**AMSTAR questions:**

Q1 – Did the research questions and inclusion criteria for the review include the components of PICO?

Q2 – Did the report of the review contain an explicit statement that the review methods were established prior to the conduct of the review and did the report justify any significant deviations from the protocol?

Q3 – Did the review authors explain their selection of the study designs for inclusion in the review?

Q4 – Did the review authors use a comprehensive literature search strategy?

Q5 – Did the review authors perform study selection in duplicate?

Q6 – Did the review authors perform data extraction in duplicate?

Q7 – Did the review authors provide a list of excluded studies and justify the exclusions?

Q8 – Did the review authors describe the included studies in adequate detail?

Q9 – Did the review authors use a satisfactory technique for assessing the risk of bias (RoB) in individual studies that were included in the review? RCTs – NRSI

Q10 – Did the review authors report on the sources of funding for the studies included in the review?

Q11 – If meta-analysis was performed did the review authors use appropriate methods for statistical combination of results? RCTs – NRSI

Q12 – If meta-analysis was performed, did the review authors assess the potential impact of RoB in individual studies on the results of the meta-analysis or other evidence synthesis?

Q13 – Did the review authors account for RoB in individual studies when interpreting/discussing the results of the review?

Q14 – Did the review authors provide a satisfactory explanation for, and discussion of, any heterogeneity observed in the results of the review?

Q15 – If they performed quantitative synthesis did the review authors carry out an adequate investigation of publication bias (small study bias) and discuss its likely impact on the results of the review?

Q16 – Did the review authors report any potential sources of conflict of interest, including any funding they received for conducting the review?

**AMSTAR domains:**

D1 – Protocol registered before commencement of the review

D2 – Adequacy of the literature search

D3 – Justification for excluding individual studies

D4 – Risk of bias from individual studies being included in the review

D5 – Appropriateness of meta-analytical methods

D6 – Consideration of risk of bias when interpreting the results of the review

D7 – Assessment of presence and likely impact of publication bias
